# Enhanced Sensitivity to Rapid Input Fluctuations by Nonlinear Threshold Dynamics in Neocortical Pyramidal Neurons

**DOI:** 10.1371/journal.pcbi.1004761

**Published:** 2016-02-23

**Authors:** Skander Mensi, Olivier Hagens, Wulfram Gerstner, Christian Pozzorini

**Affiliations:** 1 Laboratory of Computational Neuroscience (LCN), Brain Mind Institute, School of Computer and Communication Sciences and School of Life Sciences, École Polytechnique Fédérale de Lausanne, Lausanne, Switzerland; 2 Laboratory of Neural Microcircuitry (LNMC), Brain Mind Institute, School of Life Sciences, École Polytechnique Fédérale de Lausanne, Lausanne, Switzerland; University of Connecticut, UNITED STATES

## Abstract

The way in which single neurons transform input into output spike trains has fundamental consequences for network coding. Theories and modeling studies based on standard Integrate-and-Fire models implicitly assume that, in response to increasingly strong inputs, neurons modify their coding strategy by progressively reducing their selective sensitivity to rapid input fluctuations. Combining mathematical modeling with *in vitro* experiments, we demonstrate that, in L5 pyramidal neurons, the firing threshold dynamics adaptively adjust the effective timescale of somatic integration in order to preserve sensitivity to rapid signals over a broad range of input statistics. For that, a new Generalized Integrate-and-Fire model featuring nonlinear firing threshold dynamics and conductance-based adaptation is introduced that outperforms state-of-the-art neuron models in predicting the spiking activity of neurons responding to a variety of *in vivo*-like fluctuating currents. Our model allows for efficient parameter extraction and can be analytically mapped to a Generalized Linear Model in which both the input filter—describing somatic integration—and the spike-history filter—accounting for spike-frequency adaptation—dynamically adapt to the input statistics, as experimentally observed. Overall, our results provide new insights on the computational role of different biophysical processes known to underlie adaptive coding in single neurons and support previous theoretical findings indicating that the nonlinear dynamics of the firing threshold due to Na^+^-channel inactivation regulate the sensitivity to rapid input fluctuations.

## Author Summary

Over the last decades, a variety of simplified spiking models have been shown to achieve a surprisingly high performance in predicting the neuronal responses to *in vitro* somatic current injections. Because of the complex adaptive behavior featured by cortical neurons, this success is however restricted to limited stimulus ranges: model parameters optimized for a specific input regime are often inappropriate to describe the response to input currents with different statistical properties. In the present study, a new spiking neuron model is introduced that captures single-neuron computation over a wide range of input statistics and explains different aspects of the neuronal dynamics within a single framework. Our results indicate that complex forms of single neuron adaptation are mediated by the nonlinear dynamics of the firing threshold and that the input-output transformation performed by cortical pyramidal neurons can be intuitively understood in terms of an enhanced Generalized Linear Model in which both the input filter and the spike-history filter adapt to the input statistics.

## Introduction

How do pyramidal neurons transform their input into output spike trains? The answer to this question is of fundamental importance because it determines potential coding strategies of the brain [1–5]. Theoretical studies of the Integrate-and-Fire model responding to *in vivo*-like fluctuating currents concluded that, depending on the average strength *μ*_I_ (DC component) of the input current, single neurons can operate in two different regimes known as the *fluctuation driven regime* and the *mean driven regime* [6]. While in the fluctuation driven regime output spikes are exclusively evoked by transient excursions of the membrane potential (such as those caused by a volley of synchronous inputs), a neuron operating in the mean driven regime is continuously active and its output firing rate encodes the average intensity of the input current.

This view has recently been challenged by *in vitro* recordings demonstrating that the output firing rate of pyramidal neurons from rat prefrontal cortex (PFC) [7], somatosensory cortex (SSC) [8] and hippocampus [9] always increases with the amplitude *σ*_I_ of rapid input fluctuations, independent of the DC component *μ*_*I*_. These results indicate that pyramidal neurons are not static entities, but adapt their intrinsic dynamics in order to maintain selective sensitivity to rapid input fluctuations over a broad range of input statistics. Experimental evidence supporting the view that the input-output transformation performed by single neurons generally depends on the input statistics has also been provided by *in vitro* measurements of the frequency-response properties of different neuronal types [10–13]. Because of this complex adaptive behavior, it remains a major challenge to design a spiking neuron model that is at the same time simple enough to be understood from a computational perspective and flexible enough to predict spikes over an extended range of input statistics [14].

Enhanced sensitivity to rapid input fluctuations has been initially linked to slow adaptation mechanisms [7, 8, 15] and has been qualitatively reproduced *in silico* using Hodgkin-Huxley models featuring either decreased Na^+^-conductance, increased K^+^-conductance, increased leak conductance, slow Na^+^–channel inactivation or low-threshold K^+^-channels [7, 15–17]. Consistent with the fact that different biophysical mechanisms can lead to enhanced sensitivity to rapid input fluctuations, theoretical studies based on the Morris-Lecar model related this phenomenon to fundamental aspects of the spike initiation dynamics [5, 18, 19]. While these studies indicate that enhanced sensitivity to rapid input fluctuations is essentially mediated by subthreshold-activating currents [19], recent theoretical studies demonstrate that responsiveness to rapid signals can also be enhanced by a nonlinear dynamics of the firing threshold due to fast Na^+^–channel inactivation [20, 21].

In the present study, we recorded the *in vitro* response of layer 5 pyramidal (L5 Pyr) neurons to *in vivo*-like fluctuating currents of different offsets *μ*_I_ and standard deviations *σ*_I_. In agreement with previous results from distinct neuronal cell types, we found that: i) the average rate response remained sensitive to rapid input fluctuations over a broad range of offsets *μ*_I_; ii) the effective timescale of somatic integration was progressively reduced with increasing *μ*_I_; iii) the membrane potential at which spikes originated was correlated positively with *μ*_I_ and negatively with *σ*_I_. To explain these seemingly different phenomena within a single mathematical framework, we introduced a new spiking model—called the inactivating Generalized Integrate-and-Fire (iGIF) model—in which the firing threshold is nonlinearly coupled to the subthreshold membrane potential and depends linearly on the spike history. Despite its relative complexity, the iGIF model remains amenable to analytical treatment and, while being linked to known biophysical processes, its parameters can be efficiently extracted from intracellular recordings using a new likelihood-based method.

The iGIF model is first shown to capture enhanced sensitivity to rapid input fluctuations, account for firing threshold variability and predict the spiking activity of L5 Pyr neurons over an extended range of input statistics. To study the computational role of the firing threshold dynamics, the iGIF model is then analytically mapped to a Generalized Linear Model (GLM) [22, 23] in which both the input filter and the spike-history filter dynamically adapt to the input statistics. In agreement with the theoretical predictions of Platkiewicz and Brette [21] and recent experimental findings from the barn owl cochlear nucleus [24, 25], our experimental and theoretical results demonstrate that the effective timescale over which pyramidal neurons integrate their inputs is not entirely controlled by the membrane timescale, but adapts to the input statistics as a result of the nonlinear dynamics of the firing threshold. This adaptive behavior promotes detection of rapid signals over an extended range of input statistics, thus explaining enhanced sensitivity to input fluctuations in L5 Pyr neurons.

## Results

Neocortical neurons *in vivo* receive barrages of excitatory and inhibitory inputs. To understand how synaptic inputs are transformed into output spike trains, single neurons can be tested *in vitro* with somatic injections of rapidly fluctuating currents [26–29]. *In vivo*-like fluctuating currents *I*(*t*) mimic the net input received at the soma of a postsynaptic neuron. Since *in vivo* both the strength and the synchrony of afferent spike-trains can change over time, single neurons are likely to receive inputs with varying statistics [30].

### Enhanced sensitivity to rapid input fluctuations

**Fig 1:**
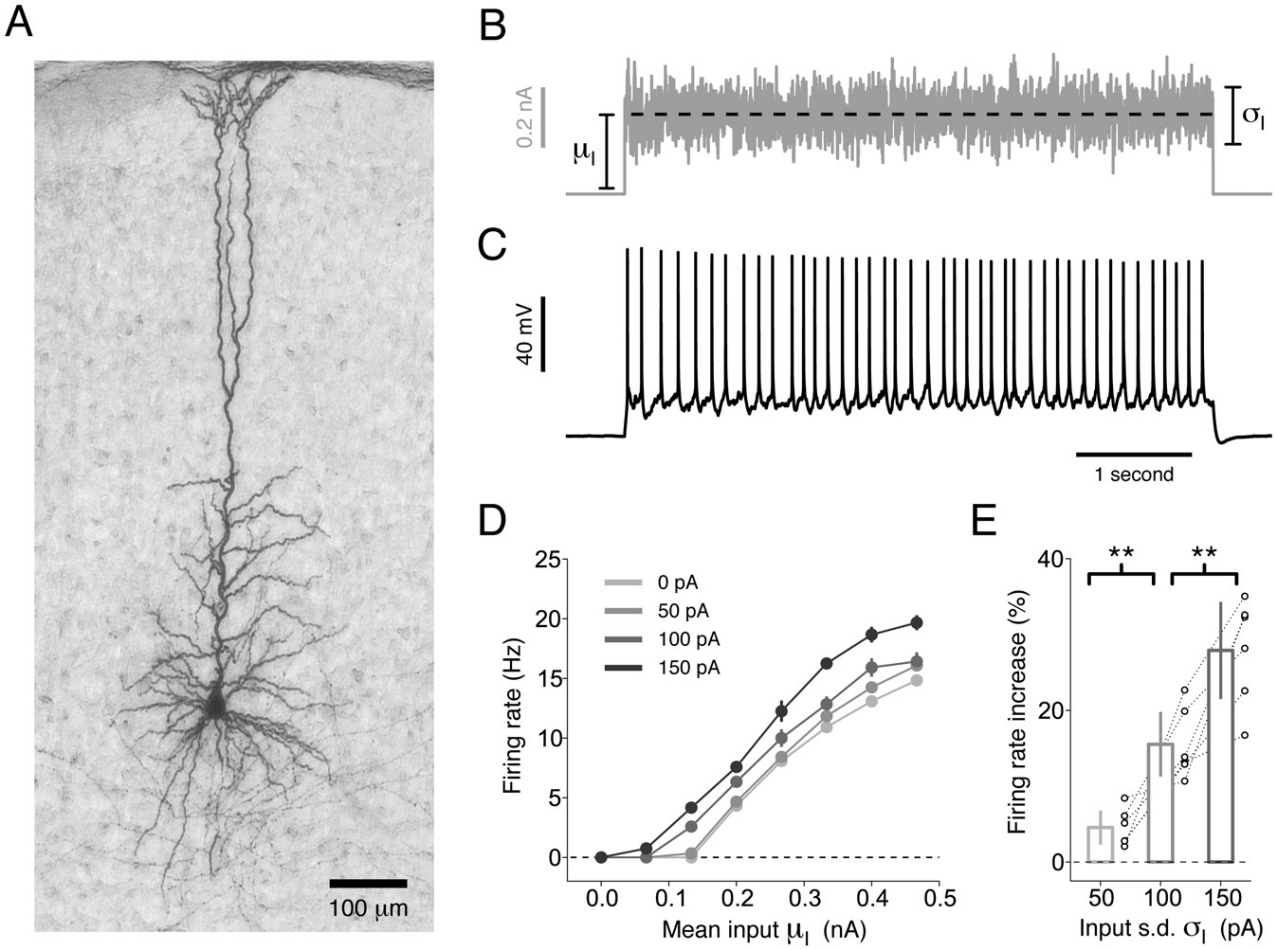
Pyr neurons maintain sensitivity to rapid input fluctuations over a wide range of depolarizing offsets. **(A)** L5 Pyr neuron stained with biocytin. **(B)** Neurons were tested with a set of 5-second fluctuating currents of different mean *μ*_*I*_ (dashed black), and magnitude of input fluctuations, *σ*_*I*_. **(C)** Typical response of a L5 Pyr neuron to a somatic injection of the current shown in panel *B* (*μ*_I_ = 0.27 nA, *σ*_I_ = 50 pA). **(D)** Steady-state firing rate *f* of a typical Pyr neuron as a function of the mean input *μ*_I_ (i.e., *f*−*μ*_I_ curve). Different lines indicate different levels of input fluctuations *σ*_I_ (see legend). For each combination of input parameters (*μ*_I_, *σ*_I_), three different 5-s current injections were performed. Firing rates were estimated during the last four seconds of the response. Error bars indicate one standard deviation across repetitions and are often too small to be visible. **(E)** Summary data obtained in different Pyr neurons (*n* = 6) by computing the percentage change in steady-state firing rate obtained by increasing the input standard deviation *σ*_*I*_ at the strongest *μ*_I_ used for each cell. Changes were computed with respect to *σ*_*I*_ = 0 pA. Each set of open circles represents data from a particular cell. Bar plots represent the mean and one standard deviation across cells. Increasing *σ*_I_ from 50 to 100 pA (*n* = 6, paired Student *t*-test, *t* = 4.4, *p* = 6.7 ⋅ 10^−3^) and from 100 to 150 pA (*n* = 6, paired Student *t*-test, *t* = 4.5, *p* = 6.5 ⋅ 10^−3^) significantly increased the firing rate.

To study single-neuron computation over a broad range of input statistics, we intracellularly recorded the response of cortical neurons evoked *in vitro* by a set of 5-second currents generated by independently varying the mean *μ*_I_ and the standard deviation *σ*_I_ of the fluctuations (Fig 1A–1C). *In vivo*-like fluctuating currents were generated with a filtered Gaussian process and injected at the soma of L5 Pyr neurons of mouse SSC (see Materials and Methods). Mimicking the activity levels observed in awake mice [31], neurons responded by emitting action potentials at rates between 0 and 20 Hz. In agreement with previous results from rat PFC [7, 32], SSC [27] and hippocampus [9], augmenting the magnitude of input fluctuations significantly increased the output firing rate over the entire range of depolarizing offsets that were tested (Fig 1D and 1E). This result is at odds with theories based on standard Integrate-and-Fire models, stating that rapid input fluctuations affect the average firing rate only in the fluctuation driven regime, where the mean input by itself is not sufficient to evoke action potentials [27, 33].

### The effective timescale of somatic integration adapts to the input statistics

**Fig 2:**
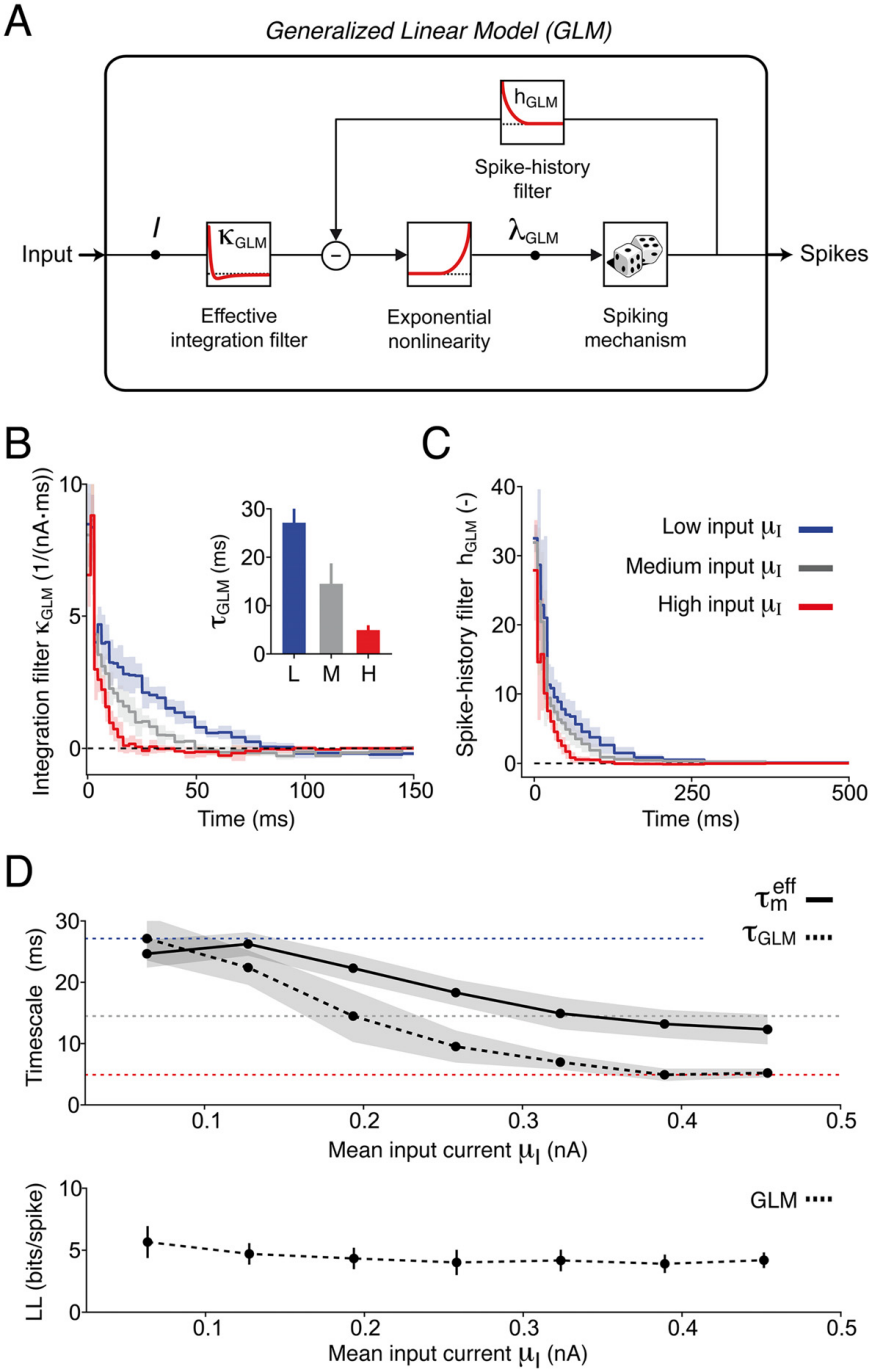
A comparison between GLM filters estimated under different input statistics reveals complex forms of single neuron adaptation. **(A)** Schematic representation of the GLM. The input current *I*(*t*) is first low-pass filtered through *κ*_GLM_(*t*) and then transformed by an exponential nonlinearity into a firing intensity λ_GLM_(*t*). Spikes are generated stochastically with rate λ_GLM_(*t*). Each time an action potential is fired, a feedback process *h*_GLM_(*t*) is triggered that accounts for spike-history dependence. **(B)-(C)** Average GLM filters *κ*_GLM_(*t*) and *h*_GLM_(*t*) extracted from 6 Pyr neurons by splitting the experimental data in eight groups according to $\mu_{\text{I}}=\left\{\mu_{\text{I}}^{\text{(1)}},\mu_{\text{I}}^{\text{(2)}},\ldots ,\mu_{\text{I}}^{\text{(8)}}\right\}$. For clarity, only three filters are shown: low input ($\mu_{\text{I}}^{\text{(2)}}=64$, s.d. 7 pA, blue), medium input ($\mu_{\text{I}}^{\text{(4)}}=190$, s.d. 30 pA, gray), strong input ($\mu_{\text{I}}^{\text{(7)}}=390$, s.d. 50 pA, red). Shaded areas indicate one standard deviation across neurons. **(B)** Average integration filters *κ*_GLM_(*t*). Inset: timescale *τ*_GLM_ of effective somatic integration quantified by fitting the filters *κ*_GLM_(*t*) with an exponential function. **(C)** Average spike-history filters *h*_GLM_(*t*). **(D)** Top: The effective timescale of somatic integration *τ*_GLM_ (dashed black) is plotted as a function of the input strength *μ*_I_ and compared to the effective membrane timescale $\tau_{\text{m}}^{\text{eff}}$ (solid black). For mean inputs *μ*_I_ ≥ 0.2 nA the effective timescale of somatic integration *τ*_GLM_ cannot be explained by conductance-increase only. The shaded areas represent one standard deviation across neurons. Colored lines represent the timescales *τ*_GLM_ extracted from the three filters shown in panel *B*. Bottom: The cross-validated GLM goodness-of-fit, as quantified by the normalized log-likelihood *LL* (see Materials and Methods), is plotted as a function of the mean input strength *μ*_I_. GLMs with input-dependent filters perform similarly across levels of input strength *μ*_I_.

To characterize the mechanisms underlying enhanced sensitivity to input fluctuations, we fitted several Generalized Linear Models (GLM, see [22, 23]) to datasets of different *μ*_I_ (see Materials and Methods). In our GLM, spikes are generated stochastically with a firing intensity that depends on the input current as well as on previous action potentials (Fig 2A). Briefly, the input current is first passed through a linear filter *κ*_GLM_(*t*) and then transformed into a firing intensity by an exponential nonlinearity. Each time an action potential is fired, an adaptation process *h*_GLM_(*t*) is triggered. In contrast to a Linear-Nonlinear-Poisson (LNP) model (which can be considered as a GLM without spike-history filter *h*_GLM_(*t*)), a GLM with spike-history filter is equivalent to a Generalized Integrate-and-Fire model [6, 34]. When LNP models are used to relate an external input (e.g., a visual input) to the spiking activity of a neuron recorded *in vivo*, the input filter—generally measured by spike-triggered averaging (STA)—is interpreted as a receptive field [35]. When a GLM with spike-history filter is used instead [23] (i.e., when non-Poissonian firing statistics are accounted for), then the input filter *κ*_GLM_(*t*) generally differs from the STA, but provides a better estimator of the neuron’s receptive field [35, 36].

Here, a GLM with spike-history filter is used to model the spiking activity of a neuron responding *in vitro* to somatic current injections [34]. In this case, *κ*_GLM_(*t*) describes the temporal window over which the neuron effectively integrates its input to generate action potentials (cf., Eq 10). Since the GLM is equivalent to a Generalized Integrate-and-Fire model [6, 34], the input filter *κ*_GLM_(*t*) is commonly interpreted as the membrane filter (that is, the filter linking the input current to the subthreshold membrane potential). However, since GLM parameter extraction entirely relies on spiking data and does not exploit the information contained in the subthreshold membrane potential fluctuations, *κ*_GLM_(*t*) can in principle also capture other mechanisms that affect the spiking probability without altering the membrane potential [34]. In the following we refer to *κ*_GLM_(*t*) as *effective integration filter* and use its timescale *τ*_GLM_ to quantify the size of the temporal window over which the input is effectively integrated to generate spikes.

While the input filters of LNP models are notoriously sensitive to input statistics (see, e.g., [36–39]), the filters of a GLM are generally thought to be more stable across a broad variety of inputs. However, by comparing the GLM filters extracted under different stimulus conditions, we found that both *κ*_GLM_(*t*) and *h*_GLM_(*t*) changed with *μ*_I_ (Fig 2B and 2C). In particular, increasing *μ*_I_ resulted in shorter integration filters (Fig 2B). We quantified this result by fitting *κ*_GLM_(*t*) with a single-exponential function and found that the effective timescale of integration *τ*_GLM_ was drastically reduced from 27.1, s.d. 3.6 ms to 4.9, s.d. 1.0 ms (Fig 2D). These results are in line with recent experimental findings from rat motorneurons [39] and suggest that L5 Pyr neurons enhance their sensitivity to rapid input fluctuations by shortening their effective timescale of integration [21]. One of the central aims of the present study is to understand why the GLM filters are input-dependent, and to relate this complex form of adaptation to the phenomenon of enhanced sensitivity to rapid input fluctuations.

Neurons can shorten their timescale of integration by increasing their total membrane conductance [40, 41]. To check whether the observed decrease in *τ*_GLM_ was due to conductance changes, we measured the effective membrane timescale $\tau_{\text{m}}^{\text{eff}}$ as a function of *μ*_I_ by fitting an Adaptive Leaky Integrate-and-Fire model to the subthreshold membrane potential fluctuations evoked by input currents with different depolarizing offsets (see Materials and Methods). Importantly, while the effective timescale of integration *τ*_GLM_ quantifies the size of the optimal filter linking the input current to output spikes (cf, Eq 10), the effective membrane timescale $\tau_{\text{m}}^{\text{eff}}$ quantifies the size of the optimal filter linking the input current to the membrane potential fluctuations. We found that changes in the effective membrane timescale only accounted for part of the reduction observed in *τ*_GLM_ (Fig 2D). We therefore have to search for an additional mechanism of timescale reduction that does not affect the subthreshold membrane potential dynamics.

### The voltage threshold for spike initiation depends on the input statistics

Previous studies indicate that enhanced sensitivity to rapid input fluctuations are mediated by a mechanism that reduces the number of Na^+^-channels available for spike initiation [7, 8, 15, 17]. In particular, a theoretical study of Platkiewicz and Brette [21] recently showed that fast Na^+^-channel inactivation could enhance sensitivity to rapid input fluctuations by making the firing threshold nonlinearly dependent on the subthreshold membrane potential. A nonlinear coupling between membrane potential and firing threshold could additionally explain the experimental discrepancy between $\tau_{\text{m}}^{\text{eff}}$ and *τ*_GLM_ [21].

**Fig 3:**
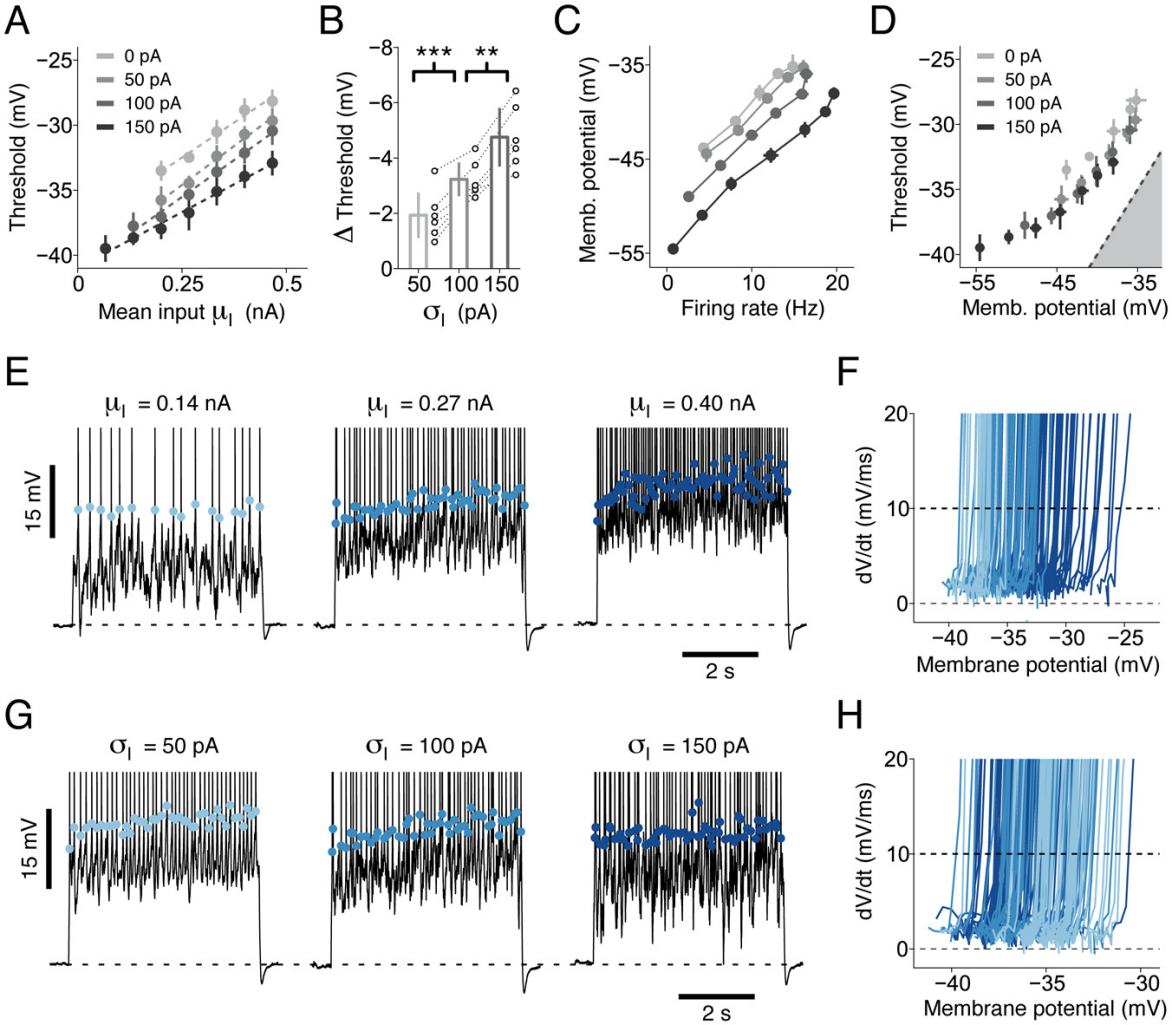
Standard analysis of intracellular recordings reveals an intricate dependence of the firing threshold on the input statistics. **(A)** Average voltage threshold for spike initiation as a function of *μ*_I_. Different gray levels indicate different levels of input fluctuations *σ*_*I*_ (see legend). Dashed lines show four linear regressions performed on experimental data with different *σ*_I_. The regression slopes *s*_LR_ observed in 6 different neurons were significantly larger than zero (*n* = 24, *s*_LR_ = 22.4, s.d. 2.7 mv/nA, Student *t*-test, *t* = 39.9, *p* < 10^−6^). **(B)** Summary data from six L5 Pyr neurons showing the relative change in voltage threshold induced by an increase in *σ*_I_. For each cell, changes were computed with respect to *σ*_I_ = 0 pA by averaging the results obtained for all depolarizing offsets *μ*_I_. Each set of open circles represents data from a particular cell. Bar plots represent mean and standard deviation across cells. Increasing *σ*_I_ from 50 to 100 pA (*n* = 6, paired Student *t*-test, *t* = 7.5, *p* = 6.8 ⋅ 10^−4^) and from 100 to 150 pA (*n* = 6, paired Student *t*-test, *t* = 6.4, *p* = 1.3 ⋅ 10^−3^) significantly reduced the voltage threshold for spike initiation. **(C)** Average mean subthreshold membrane potential as a function of the average firing rate. Color code as in panel *A*. **(D)** Average voltage threshold as a function of the mean subthreshold membrane potential. Different gray levels indicate different *σ*_I_ (see legend). The dashed line shows the identity diagonal delimitating the area (gray) in which the membrane potential is larger than the firing threshold. **(E)-(F)** The firing threshold is positively correlated with *μ*_I_. **(E)** Intracellular recordings obtained with three different values of *μ*_I_ (and *σ*_I_ = 100 pA). Colored dots indicate the membrane potential at spike initiation. Different colors denote different input statistics. For clarity the y-axis was truncated at -20 mV. **(F)** For each action potential shown in panel *E*, the voltage derivative *dV*_data_/*dt* is plotted as a function of the membrane potential *V*_data_. The voltage threshold for spike initiation was defined as the voltage where *dV*_data_/*dt* crossed 10 mV/ms from below (dashed black). Different colors indicate different values of *μ*_I_ (color code as in panel *E*). **(G)-(H)** The firing threshold is negatively correlated with *σ*_I_. The same analysis as in panels *E-F* was performed on intracellular recordings obtained in response to three values of *σ*_I_ (and *μ*_I_ = 0.2 nA). Different colors indicate different values of *σ*_I_. In panels *A*, *C* and *D*, each data point was computed by averaging the results obtained by analyzing the responses to three different 5-second currents with the same input statistics (*μ*_*I*_, *σ*_*I*_). Consequently, error bars indicate one standard deviation across repetitions. Data are from the same cell shown in Fig 1. As in Fig 1, action potentials recorded in the first second of each repetition were not considered. Since voltage recordings were not corrected for the liquid junction potential, all membrane potentials and firing thresholds reported in this figure are positively biased by approximately 14.5 mV (see Materials and Methods).

Using standard methods, we extracted from intracellular recordings the voltage at which individual action potentials were initiated (see Materials and Methods). Consistent with earlier results [41–44], the voltage threshold for spike initiation always increased with the mean input *μ*_I_ (Fig 3A, 3E and 3F) and was reduced in the presence of input fluctuations *σ*_I_ (Fig 3A, 3B, 3G and 3H). By further analyzing our raw data, we found that at a given firing rate, the average subthreshold membrane potential always decreased with increasing levels of input fluctuations (Fig 3C). Consistent with the hypothesis that the firing threshold reduction observed under large input fluctuations was mediated by a firing threshold dependence on the subthreshold membrane voltage [21, 43], we found that, on average, these two quantities were nonlinearly related (Fig 3D). Overall, the results reported in Fig 3 confirm previous experimental findings and are in line with the theoretical predictions of Platkiewicz and Brette [21].

In the next section, a new Generalized Integrate-and-Fire model is introduced that will allow us to provide direct evidence for a nonlinear coupling between membrane potential and firing threshold. By analyzing the model, we will then explain: i) why the voltage threshold for spike initiation depends on the input statistics (Fig 3); ii) why the effective timescale of somatic integration is shorter than the membrane timescale and adapts to the input statistics (Fig 2); iii) how L5 Pyr neurons maintain sensitivity to rapid input fluctuations over a broad range of input statistics (Fig 1).

### Inactivating Generalized Integrate-and-Fire (iGIF) model

**Fig 4:**
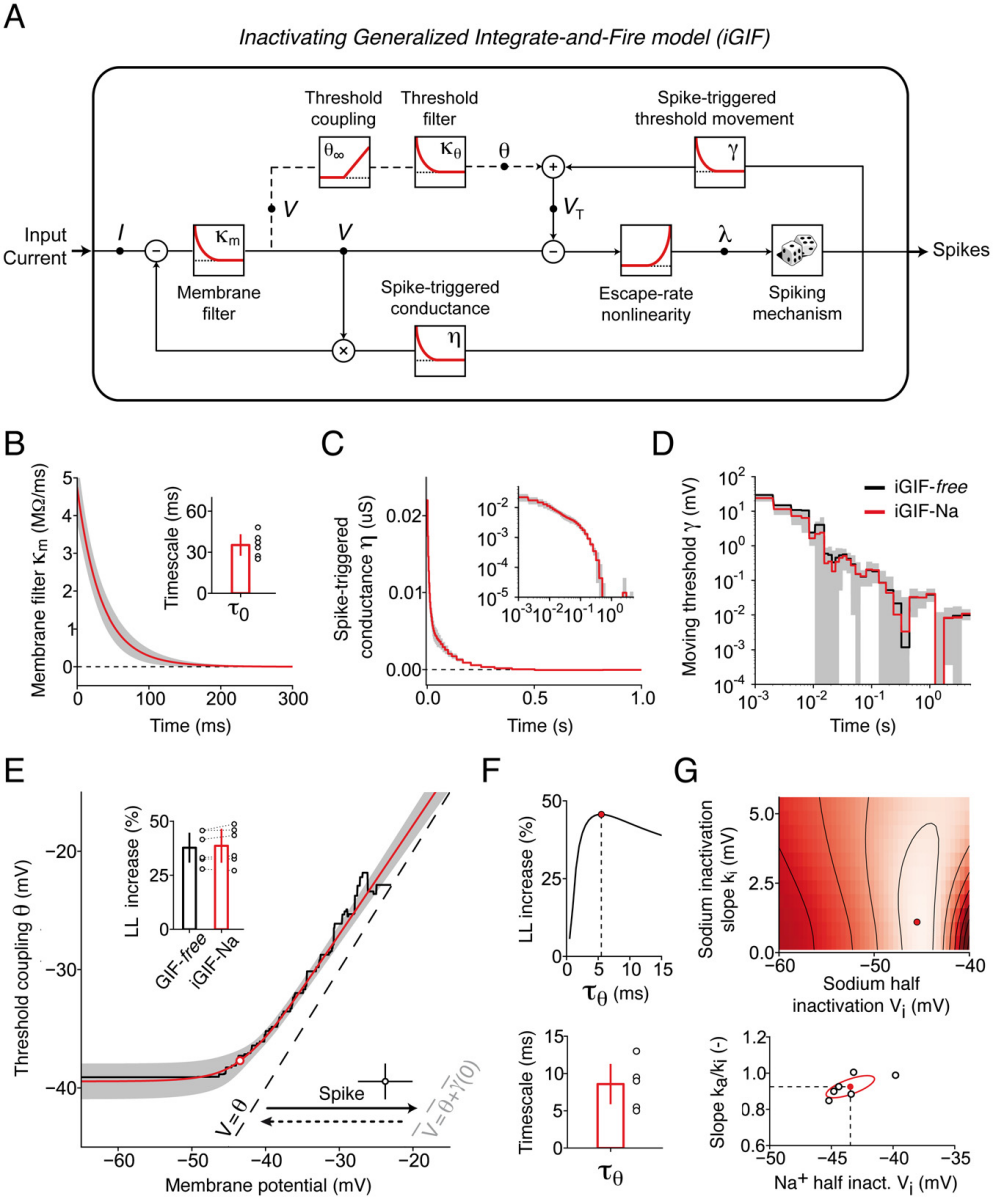
iGIF model with parameters extracted from intracellular recordings. **(A)** Schematic representation of the iGIF model. The input current is first low-pass filtered by the *Passive membrane filter* $\kappa_{\text{m}}\left(t\right)=\Theta \left(t\right)C^{-1}e^{-\frac{t}{\tau_{\text{m}}}}$. The resulting signal models the subthreshold membrane potential *V*(*t*) and, after subtraction of the firing threshold *V*_T_(*t*), is transformed into a firing intensity λ(*t*) by the exponential *Escape-rate nonlinearity*. Spikes are emitted stochastically and elicit both a *Spike-triggered conductance* *η*(*t*) and a *Spike-triggered threshold movement* *γ*(*t*). In the iGIF model, but not in the GIF model, the firing threshold *V*_T_(*t*) is coupled to the subthreshold membrane potential (dashed circuit). For that, the membrane potential *V*(*t*) is first passed through the nonlinear *Threshold coupling* function *θ*_∞_(*V*) and then low-pass filtered by the *Threshold filter* $\kappa_{\theta }\left(t\right)=\Theta \left(t\right)\tau_{\theta }^{-1}e^{-\frac{t}{\tau_{\theta }}}$. **(B)-(E)** Average parameters extracted from 6 Pyr neurons. Black: iGIF-free, red: iGIF-Na. Gray areas indicate one standard deviation across cells for the iGIF-Na model. **(B)** Passive membrane filter *κ*_m_(*t*). Inset: passive membrane timescale *τ*_m_. Open circles: results from individual cells. Bar plot: mean and standard deviation. **(C)** Spike-triggered conductance *η*(*t*). Inset: same data on log-log scales. **(D)** Spike-triggered threshold movement *γ*(*t*). **(E)** Nonlinear threshold coupling *θ*_∞_(*V*) (iGIF-free, solid black line; iGIF-Na, solid red line). Spikes are emitted stochastically when the spiking boundary *V* = *V*_T_ is approached. This boundary defines the line where the probability *p* of emitting a spike during a time bin of Δ*T* = 0.1 ms reaches *p* = 0.63. To the right, the probability increases further. In the absence of previous action potentials, the spiking boundary is given by *V* = *θ* (dashed black). After an action potential, the spiking boundary instantaneously shifts to the right by *γ*(0)≈25 mV (dashed gray), and then slowly decays back to *V* = *θ*. After each spike, the variables (*V*(*t*), *θ*(*t*)) are reset to $\left(V_{\text{reset}},V_{T}^{*}\right)$ (open circle; error bars denote one standard deviation across cells). The open red circle indicates the Na^+^ half-inactivation voltage *V*_i_, where the threshold becomes sensitive to the membrane potential. Inset: percentage increase in model log-likelihood (*LL*) of iGIF models with respect to the GIF model. Open circles: model performance on the same cell for iGIF-free and iGIF-Na. Bar plots: mean and standard deviation across neurons. **(F)** Top: *LL* percentage increase of the iGIF-free model as a function of *τ*_*θ*_. Red circle: optimal timescale *τ*_*θ*_. Data are shown from a typical neuron. Bottom: optimal timescales *τ*_*θ*_ extracted from 6 Pyr neurons. **(G)** Top: *LL* percentage increase of the iGIF-Na model as a function of *k*_*i*_ and *V*_*i*_. The *LL* increases from dark to light red. Red circle: optimal parameters. Bottom: optimal parameters extracted from 6 different Pyr neurons. Note that the half-inactivation voltages *V*_*i*_ reported in the figure are positively biased due to uncompensated liquid junction potential of 14.5 mV (see Materials and Methods). The mean and the standard deviational ellipse across cells are shown in red.

To explain within a single framework the experimental findings reported in Figs 1–3, we fitted a new spiking model to intracellular recordings. The model was obtained by extending our previous Generalized Integrate-and-Fire model (GIF; [44, 45]) with a nonlinear coupling between firing threshold and membrane potential, which possibly arises from fast Na^+^-channel inactivation [21]. We refer to this model as iGIF, where *i* stands for *inactivating* (Fig 4A, see Materials and Methods). In the iGIF model, spikes are generated stochastically according to a firing intensity which exponentially depends on the instantaneous difference between the membrane potential *V* and firing threshold *V*_T_ [29, 46]:

$$\lambda =\lambda_{0}\exp\left(\frac{V-V_{\text{T}}}{\Delta V}\right),$$ (1)

where λ_0_ = 10 kHz is a constant and the parameter Δ*V* regulates the level of stochasticity. When Δ*V* → 0, the model becomes deterministic and spikes are emitted reliably whenever the voltage reaches the firing threshold. When Δ*V* > 0, the membrane potential can cross the firing threshold without emitting a spike and spikes can be emitted even when the membrane potential is below threshold. Consequently, when Δ*V* is large, the iGIF model features strong trial-to-trial variability and generates action potentials with poor temporal precision.

The dynamics of the membrane potential are deterministic and modeled as a leaky integrator augmented with an adaptation current *I*_A_:

$$C\dot{V}=-g_{\text{L}}\left(V-E_{\text{L}}\right)+I-I_{\text{A}},$$ (2)

where *I* denotes the input current, *C*, *g*_L_ and *E*_L_ define the passive properties of the membrane and the dynamics of the adaptation current *I*_A_ are described by the following conductance-based model [47]:

$$I_{\text{A}}\left(t\right)=\sum_{{\hat{t}}_{j}<t}\eta \left(t-{\hat{t}}_{j}\right)\cdot \left(V-E_{\text{R}}\right),$$ (3)

where *E*_R_ is a reversal potential and $\eta (t-{\hat{t}}_{j})$ describes the time course of the conductance change triggered by the emission of an action potential at time ${\hat{t}}_{j}$.

The dynamics of the firing threshold *V*_T_ can be derived mathematically from an Hodgkin-Huxley model featuring fast and slow Na^+^-channel inactivation (see refs. [21] and Materials and Methods) and writes:

$$V_{\text{T}}\left(t\right)=\theta \left(t\right)+\sum_{{\hat{t}}_{j}<t}\gamma \left(t-{\hat{t}}_{j}\right),$$ (4)

where *γ*(*t*) describes threshold changes induced by a previous action potential and *θ*(*t*) implements a nonlinear coupling between *V*_*T*_ and *V*, which is governed by the differential equation [21]:

$$\tau_{\theta }\dot{\theta }\left(t\right)=-\theta \left(t\right)+V_{\text{T}}^{*}+\theta_{\infty }\left(V\right),$$ (5)

with $V_{\text{T}}^{*}$ being the threshold baseline. Depending on the functional shape of the steady-state function *θ*_∞_(*V*), Eq 5 can make the firing threshold sensitive to the depolarization rate of the membrane potential [21, 48]. Indeed, if *θ*_∞_(*V*) is an increasing function of *V*, membrane depolarizations occurring on a slower rate than the characteristic timescale *τ*_*θ*_ will raise the firing threshold. Consequently, compared to fast inputs, slow currents will evoke action potentials that are initiated at larger voltages [21, 48]. The threshold mechanism described in Eq 5 can thus shorten the effective timescale over which the input current is integrated to generate spikes, without affecting the subthreshold voltage dynamics [21, 24].

Importantly, the functional shape of *η*(*t*), *γ*(*t*) and *θ*_∞_(*V*), along with all the other iGIF model parameters, are extracted from intracellular recordings using a two-step fitting procedure (see Materials and Methods; a Python implementation is freely available at https://github.com/pozzorin/GIFFittingToolbox). In the first step, the parameters governing the subthreshold dynamics of the membrane potential—including the functional shape of the spike-triggered conductance *η*(*t*)—are extracted by minimizing the sum of squared errors on the subthreshold voltage derivative. In the second step, the parameters governing the dynamics of the firing threshold—including the functional shape of the voltage-coupling *θ*_∞_(*V*) and the spike-history filter *γ*(*t*)—are obtained with a maximum likelihood approach similar to the one used to fit GLMs to spiking data [22, 23]. In what follows, we refer to the iGIF model estimated using this method as iGIF-*free*, where *free* indicates that the functional shape of the above-mentioned functions is not defined *a priori*, but is extracted from data.

### iGIF model parameters extracted from intracellular recordings reveal a nonlinear coupling between membrane potential and firing threshold

We found that the passive properties of the membrane were characterized by a timescale *τ*_m_ = 35.3, s.d. 8.6 ms (Fig 4B). Spike-triggered conductance changes were always positive (Fig 4C) and associated with a low reversal potential *E*_R_ = -57.0, s.d. 3.9 mV. When displayed on log-log scales, the decay of the spike-triggered threshold movement *γ*(*t*) was approximatively linear over several orders of magnitude (Fig 4D). This result is in agreement with a previous finding that, in Pyr neurons, spike-frequency adaptation does not have a preferred timescale, but is characterized by a power-law decay [45, 49]. By extracting the shape of *θ*_∞_(*V*) directly from intracellular recordings, we found that firing threshold and subthreshold membrane potential were indeed nonlinearly coupled (Fig 4E, black). Moreover, the nonparametric estimate of *θ*_∞_(*V*) was in striking agreement with the smooth-linear rectifier predicted by a systematic reduction of the Hodgking-Huxley model [21]. This result indicates the threshold-voltage coupling arises from fast Na^+^-channels inactivation.

Since the value of the coupling timescale *τ*_*θ*_ = 8.6, s.d. 3.0 ms (Fig 4F) was also consistent with previous measurements of fast Na^+^-channel inactivation (see, e.g., ref. [50]), we used the intracellular recordings to fit a simpler iGIF model, referred to as iGIF-Na, in which *θ*_∞_(*V*) was assumed *a priori* to be the smooth linear rectifier function $\theta_{\infty }^{\text{Na}}\left(V\right)$ predicted in ref. [21]:

$$\theta_{\infty }^{\text{Na}}\left(V\right)=k_{\text{a}}\log\left(1+\exp\left(\frac{V-V_{\text{i}}}{k_{\text{i}}}\right)\right),$$ (6)

where *V*_i_ is the half-inactivation voltage of Na^+^-channels, and where the asymptotic slope of $\theta_{\infty }^{\text{Na}}\left(V\right)$ is determined by the ratio between the activation slope *k*_a_ and the inactivation slope *k*_i_ of Na^+^-channels (see Materials and Methods). Note that, in the iGIF-Na model, the firing threshold is effectively coupled to the membrane potential only when the subthreshold voltage *V* is close to or larger than *V*_i_. Moreover, an asymptotic slope *k*_a_/*k*_i_ equal or close to 1 implies that, at large voltages *V* ≫ *V*_i_, the spiking probability becomes independent of the average value of the membrane potential and is only affected by voltage fluctuations which are fast compared to the characteristic timescale *τ*_*θ*_ [21].

Both the spike-triggered movement of the firing threshold *γ*(*t*) (Fig 4D, red) and the nonlinear coupling $\theta_{\infty }^{\text{Na}}\left(V\right)$ (Fig 4E, red) extracted by fitting the iGIF-Na model to data confirmed the results obtained with the nonparametric (i.e., *free*) method (Fig 4D and 4E, black). In agreement with the fact that Na^+^-channels expressed in central neurons have similar activation and inactivation slopes (i.e., *k*_a_ ≈ *k*_i_) [21], the asymptotic slope of $\theta_{\infty }^{\text{Na}}\left(V\right)$ was very close to 1 (Fig 4G). Again in line with previous biophysical measurements [21], the half-inactivation voltage *V*_i_ obtained by taking into account the fact that recordings were not corrected for the liquid junction potential of 14.5 mV (see Materials and Methods) were comprised between -60 and -55 mV (Fig 4G).

Despite the smaller number of parameters compared to the *free* search, the log-likelihood of the iGIF-Na model was not significantly different from that of the iGIF-*free* model (Fig 4E, inset). This result provides additional evidence for the hypothesis that the biophysical mechanism underlying the nonlinear coupling between firing threshold and membrane potential is fast Na^+^-channel inactivation. In the followings, we just work with the iGIF-Na model, which, for simplicity, will be referred to as iGIF model.

### The iGIF model captures enhanced sensitivity to rapid input fluctuations and predicts spikes with millisecond precision

**Fig 5:**
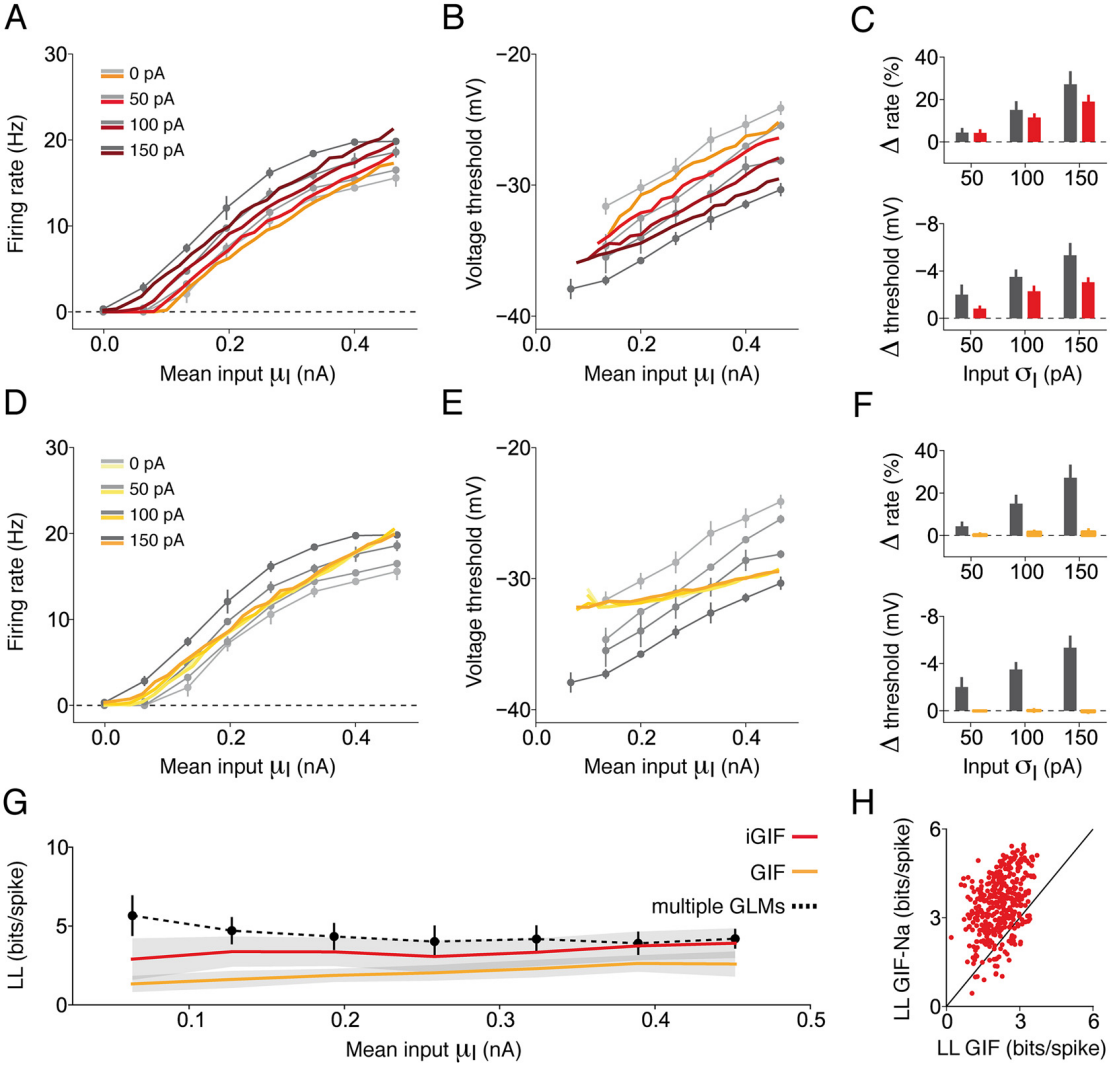
The iGIF model captures enhanced sensitivity to input fluctuations. **(A)** Comparison between steady-state *f* − *μ*_I_ curves observed in a typical Pyr neuron (gray lines) and produced by the iGIF model (colored lines). Different colors and gray levels indicate the magnitude of input fluctuations *σ*_I_ (see legends). **(B)** Average firing threshold observed in a typical cell and produced by the iGIF model. Conventions as in panel *A*. Since our threshold analysis is based on relative changes rather than on absolute values (Materials and Methods), model predictions were normalized with a constant offset to have the same mean as the experimental data. **(C)** Summary data of results obtained in six Pyr neurons (gray) and iGIF models (red). Top: percentage change in steady-state firing rate obtained in response to the strongest DC input by increasing the input standard deviation *σ*_I_ (data are presented as in Fig 1E). Bottom: average change in voltage threshold obtained by increasing the input standard deviation *σ*_I_ (data are presented as in Fig 3B). **(D)–(F)** As a control, the GIF model response (yellow) is compared against data (gray). Results are presented as in panels *A*–*C*. **(G)** The normalized, cross-validated log-likelihood *LL* (see Materials and Methods) of the iGIF (red) and the GIF model (yellow), is shown as a function of *μ*_I_. Solid lines and gray areas indicate the average and one standard deviation across neurons, respectively. For comparison, the performance of the GLM with input-specific filters is shown in black (data reported from Fig 2D). **(H)** Normalized, cross-validated log-likelihood *LL* of the iGIF model as a function of that of the GIF model. Each data point indicates the *LL* obtained from an individual neuron responding to a specific input (*μ*_I_; *σ*_I_)

To verify whether the iGIF model was able to capture enhanced sensitivity to rapid input fluctuations, we repeated our experimental paradigm *in silico* by testing the iGIF model with a set of 5-second currents generated by systematically varying the input parameters *μ*_I_ and *σ*_I_ (Fig 5A–5C). We compared the average firing rate response of the model against experimental data and found that, despite its relative simplicity, the iGIF model captured the behavior of Pyr neurons over a broad range of input statistics (Fig 5A). In particular, the iGIF model exhibited enhanced sensitivity to input fluctuations throughout the entire set of depolarizing currents *μ*_I_ that were tested and reproduced the average firing rate response with an accuracy of *ϵ*_rate_ = 1.0, s.d. 0.2 Hz (defined as the root-mean-square error between model and data). Notably, the iGIF model also captured the complex dependence of the firing threshold on input statistics. In particular, the voltage threshold at which spikes were initiated was positively correlated with *μ*_I_ (Fig 5B) and negatively correlated with *σ*_I_ (Fig 5B and 5C).

To appreciate the importance of modeling the nonlinear coupling between firing threshold and membrane potential, we also fitted our previous GIF model [44, 45] to the same experimental data. The GIF model differs from the iGIF model simply because its firing threshold dynamics only depend on the spike-history and not on the membrane potential (see Materials and Methods). As expected, the GIF model could not capture the firing rate dependence on *σ*_I_, was less accurate in reproducing the firing rates observed at steady-state (*ϵ*_rate_ = 1.7, s.d. 0.3 Hz; see Fig 5D and 5F) and was unable to explain the firing threshold dependence on the input statistics (Fig 5E and 5F). Finally, the results obtained by computing the cross-validated log-likelihood (see Materials and Methods) of the iGIF and the GIF model as a function of the input strength *μ*_I_ confirmed that a spiking model in which the firing threshold dynamics simply depend on previous action potentials is not sufficient to capture the spiking activity of Pyr neurons over a wide range of input statistics (Fig 5G and 5H).

**Fig 6:**
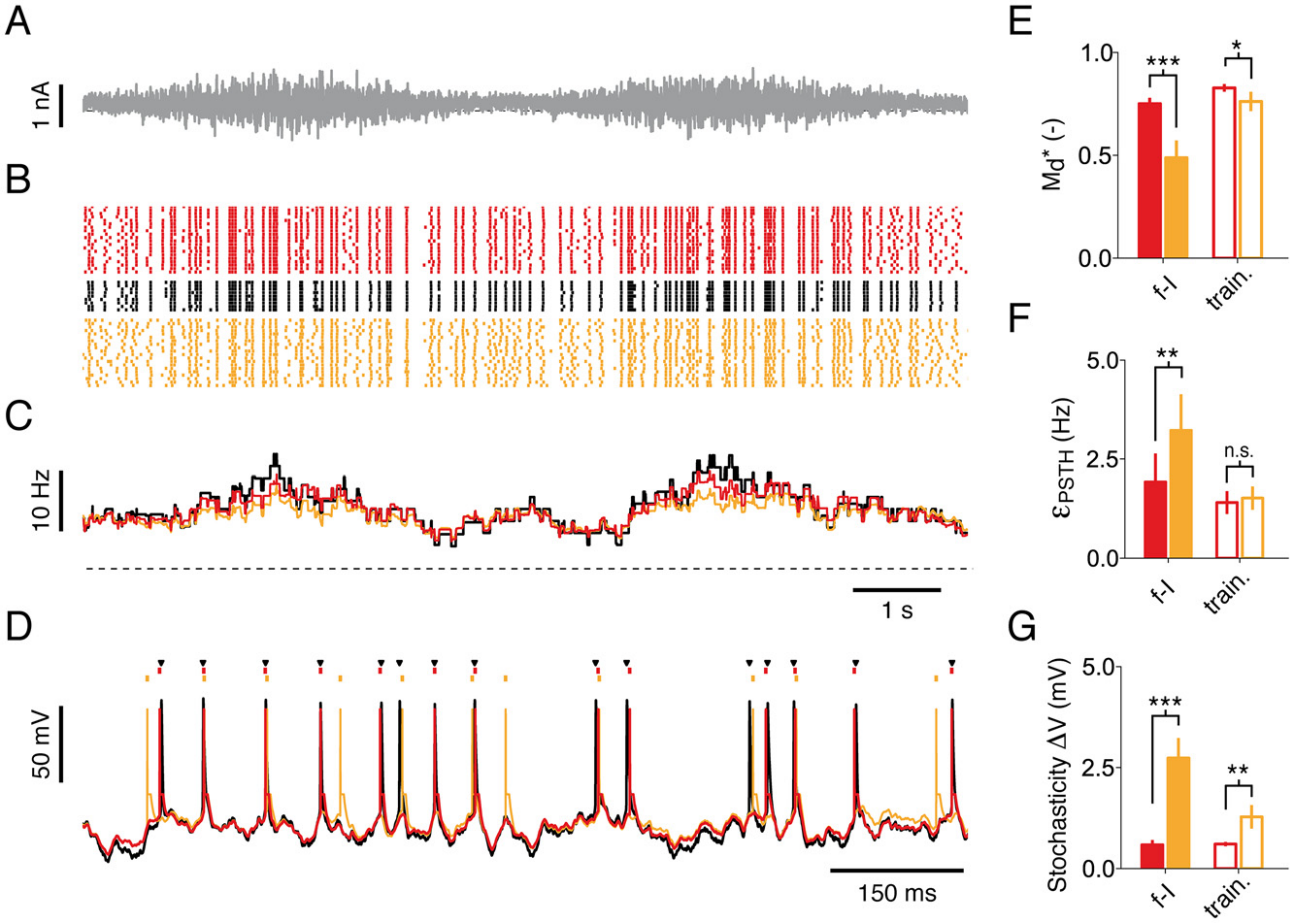
The iGIF model predicts spikes with millisecond precision. **(A)** Segment of the 20-second current used in the *test dataset*. **(B)** Spiking response of a Pyr neuron (black) to 9 repetitive injections of the current shown in panel *A*. The predictions of the iGIF and GIF model are shown in red and yellow, respectively. **(C)** PSTHs computed by filtering the spike trains shown in panel *B* with a 500 ms rectangular window. The dashed line indicates 0 Hz. **(D)** Typical intracellular response (black), as well as typical iGIF (red) and GIF (yellow) model prediction, to a single presentation of a 1-s segment of the current shown in panel *A*. Black triangles and colored dots indicate spikes. **(E)**–**(G)** Summary data showing the performance of the iGIF (red) and the GIF (yellow) model in predicting the *test dataset*. Filled bars and open bars show the performance of models trained on the *f*–*I* *dataset* and the *training dataset*, respectively. Error bars represent one standard deviation across neurons. **(E)** Spike-timing prediction as quantified by the similarity measure $M_{d}^{*}$. The iGIF model significantly outperforms the GIF model with parameters extracted from the *f*–*I* *dataset* ($M_{d}^{*}=0.75$, s.d. 0.03, iGIF; $M_{d}^{*}=0.49$, s.d. 0.08, GIF; *n* = 6, paired Student *t*-test, *t*_5_ = −8.44, *p* = 3.8 · 10^−4^) and from the *training dataset* ($M_{d}^{*}=0.83$, s.d. 0.02, iGIF; $M_{d}^{*}=0.76$, s.d. 0.05, GIF; *n* = 6, paired Student *t*-test, *t*_5_ = −3.25, *p* = 0.022). **(F)** The prediction error *ϵ*_PSTH_ on the PSTH (see panel *C*) was quantified by computing the root mean square error between data and model prediction. The iGIF model significantly outperforms the GIF model with parameters extracted from the *f*–*I* *dataset* (*ϵ*_PSTH_ = 1.93, s.d. 0.71 Hz, iGIF; *ϵ*_PSTH_ = 3.22, s.d. 0.91 Hz, GIF; *n* = 6, paired Student *t*-test, *t*_5_ = 6.33, *p* = 1.5 · 10^−3^) but not when the parameters are extracted from the *training dataset* (*ϵ*_PSTH_ = 1.41, s.d. 0.28 Hz, iGIF; *ϵ*_PSTH_ = 1.52, s.d. 0.30 Hz, GIF; *n* = 6, paired Student *t*-test, *t*_5_ = 0.95, *p* = 0.38). **(G)** Comparison between GIF the iGIF model stochasticity. The iGIF model is significantly less stochastic than the GIF model (Δ*V* = 0.59, s.d. 0.12 mV, iGIF; Δ*V* = 2.74, s.d. 0.50 mV, GIF; *n* = 6, paired Student *t*-test, *t*_5_ = 9.68, p = 2.0 · 10^−4^, *f*–*I* *dataset*; Δ*V* = 0.60, s.d. 0.05 mV, iGIF; Δ*V* = 1.28, s.d. 0.29 mV, GIF; *n* = 6, paired Student *t*-test, *t*_5_ = 5.71, p = 2.3 · 10^−3^, *training dataset*).

Beyond the firing rates, the iGIF model also reproduces the fine temporal structure of the spiking response (Fig 6), one of the aims of single-neuron modeling [51]. To take into account the fact that single neurons are stochastic [52] and to avoid problems related to overfitting, we assessed spike-timing prediction on a new experimental dataset (*test dataset*). This dataset was collected by performing nine repetitive injections of a new fluctuating current *I*_test_(*t*) (frozen-noise) that was not used for parameter extraction. In order to test the model’s ability of predicting spikes evoked by different levels of input fluctuations, the standard deviation of the current *I*_test_(*t*) was modulated by a slow sinusoidal function (Fig 6A, see Materials and Methods). On average, the iGIF model with parameters extracted from the dataset used to compute the *f*−*μ*_I_ curves (*f-I dataset*, see Fig 1) was able to predict 75.1, s.d. 3.2% of the spikes with a precision of ±4 ms (Fig 6B and 6E). The iGIF model performed well also in predicting the slow fluctuations of the firing rate induced by the sinusoidal input modulation (Fig 6C and 6F) as well as the rapid dynamics of the subthreshold membrane potential (Fig 6D). As expected, the performance achieved by the GIF model were significantly lower with 48.8, s.d. 9.2% of predicted spikes (Fig 6B–6F).

In previous studies [44, 45], we found that the GIF model was able to predict around 80% of the spikes observed in Pyr neurons responding to quasi-stationary inputs. At first glance, the low performance achieved here might therefore seem surprising. This result can however be understood by comparing the degree of stochasticity of the GIF model and the iGIF model (Fig 6G). In both models, the parameter Δ*V* regulates the level of stochasticity of the spiking process (see Eq 1). In the ideal case of a perfect model, Δ*V* is optimally tuned to capture trial-to-trial variability. In reality, a lack of flexibility in the model can bias the estimation of Δ*V* towards large values. The reason for this is that, in an oversimplified model, signals mediated by those single-neuron features that the model cannot describe are interpreted as noise [53]. While the level of stochasticity observed in the iGIF model was weak (Δ*V* = 0.59 mV, s.d. 0.13 mV), the values obtained by fitting the GIF model to the *f-I dataset* were always very high (Δ*V* = 2.74 mV, s.d. 0.54 mV), indicating that the GIF model is not sufficiently flexible to capture neuronal activity over a broad range of input statistics. Consequently, the GIF model emitted spikes with low temporal precision and achieved a poor performance in predicting individual spikes.

To make sure that the success of the iGIF model does not simply result from the aberrant level of stochasticity in the GIF model, we reassessed spike-timing prediction in both models by extracting parameters from a third dataset (*training dataset*, see Materials and Methods) obtained by injecting a 120-s current having the same statistics as the *test dataset* (Fig 6E–6G, open bars). Indicating that the GIF model is sufficiently flexible to describe the neuronal activity evoked by simpler stimuli (i.e., by stimuli that are similar to those used in our previous studies [44, 45]), its level of stochasticity dramatically decreased (Fig 6G), leading to a spike prediction reliability of $M_{\text{d}}^{*}=76.2\%$, s.d. 5.1%. Nevertheless, the iGIF model with parameters extracted from the *training dataset* still outperformed the GIF model by predicting 82.8% of spikes ($M_{\text{d}}^{*}=82.8\%$, s.d. 2.0%; Fig 6E).

Overall, these results demonstrate that the iGIF model is an excellent spiking neuron model capable of predicting individual spikes with millisecond precision and capturing the activity of Pyr neurons over a wide range of input statistics. But one central question remains unsolved: How do Pyr neurons adapt their effective timescale of integration to maintain sensitivity to rapid input fluctuations, regardless of *μ*_I_?

### Enhanced sensitivity to input fluctuations results from an interaction between spike-dependent and voltage-dependent threshold adaptation

**Fig 7:**
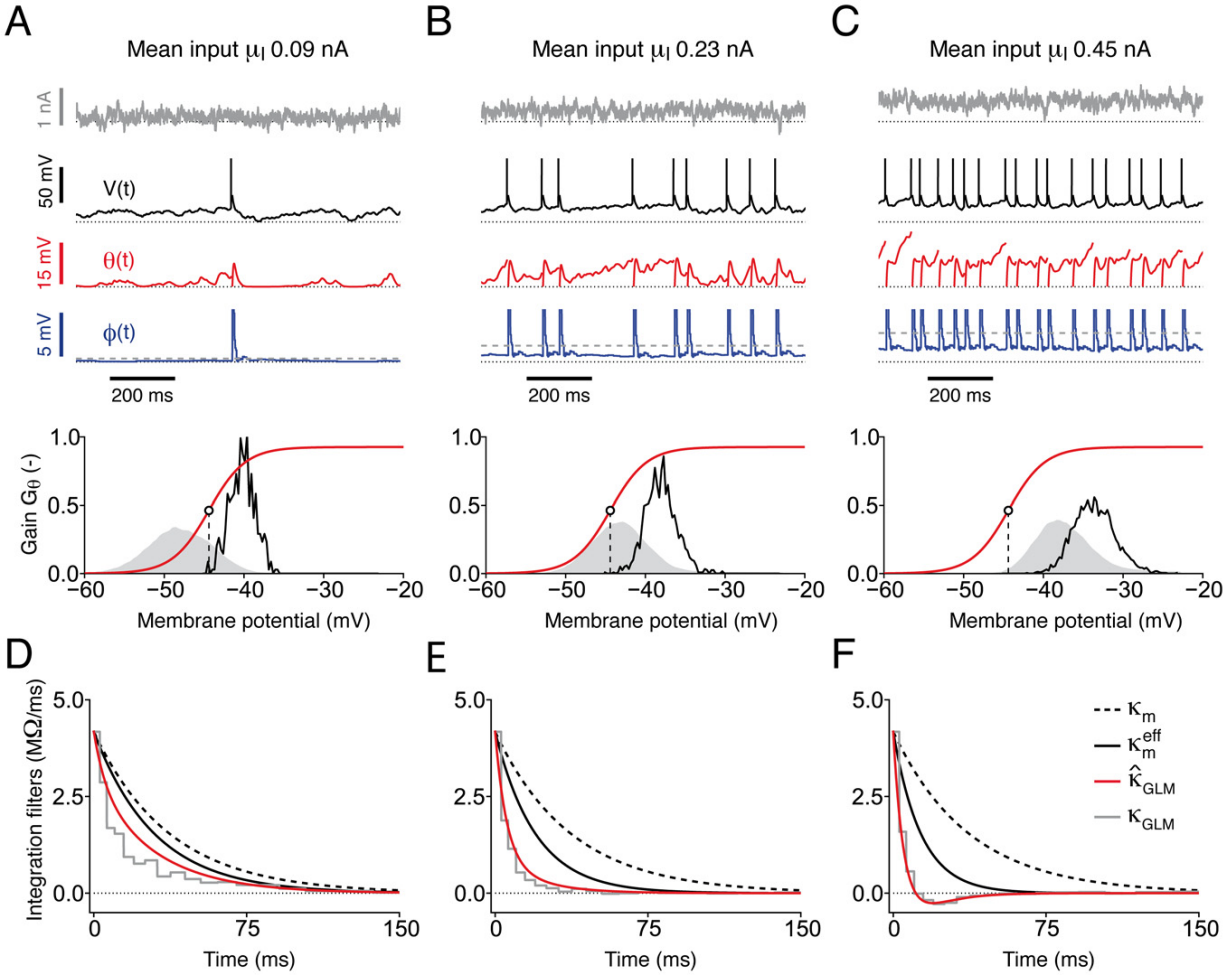
The threshold-voltage coupling progressively activates with increasing input strengths *μ*_I_. Dynamics of the iGIF model (with parameters extracted from a typical Pyr neuron) responding to three fluctuating currents with standard deviation *σ*_I_ = 100 pA, temporal correlation *τ*_I_ = 3 ms and *μ*_I_ = 90 pA (panels *A* and *D*), *μ*_I_ = 230 pA (panels *B* and *E*) and *μ*_I_ = 450 pA (panels *C* and *F*). **(A)** Top: input current *I*(*t*) (gray), membrane potential *V*(*t*) (black), voltage-dependent threshold adaptation *θ*(*t*) (red) and spike-dependent threshold adaptation $\phi \left(t\right)=\sum_{\hat{t}}\gamma \left(t-\hat{t}\right)$ (blue) as a function of time. The four dotted lines indicate: *I* = 0 nA, *V* = *E*_L_, $\theta =V_{\text{T}}^{*}$ and *φ* = 0 mV. Bottom: distribution of subthreshold membrane potential fluctuations *P*(*V*) (gray) and of voltages at which spikes were initiated *P*(*V*|spike) (black). The instantaneous threshold-coupling gain $G_{\theta }\left(V\right)=\frac{d}{dV}\theta_{\infty }^{\text{Na}}\left(V\right)$ (red) defines the sensitivity of the firing threshold to the membrane potential and can be interpreted as an activation function for the threshold-voltage coupling. Since $\theta_{\infty }^{\text{Na}}\left(V\right)$ is a nonlinear function of *V*, *G*_*θ*_ depends on the membrane potential. For low inputs, the histogram of voltage s *P*(*V*) covers mostly the region *V* ≤ *V*_*i*_ (dashed horizontal line), where the threshold-voltage coupling is not active. **(B)** Same results as in panel *A* obtained by increasing the mean input *μ*_I_. With medium input strength, the majority of the voltage distribution lies in the region *V* ≈ *V*_*i*_, where the threshold-voltage coupling is active. **(C)** As in panel *B*, but for strong mean input *μ*_I_. In this case, the voltage-threshold coupling is almost always active (i.e., *V* ≥ *V*_*i*_). **(D)** The GLM integration filter *κ*_GLM_(*t*) (solid gray) obtained by fitting a GLM to the spiking activity of the iGIF model (panel *A*) is compared against the passive membrane filter *κ*_m_(*t*) (dashed black, see Fig 4B) and the effective membrane filter $\kappa_{\text{m}}^{\text{eff}}\left(t\right)$ (solid black), which accounts for the conductance increase mediated by *η*. The GLM integration filter *κ*_GLM_(*t*) (solid gray) matches the theoretical filter ${\hat{\kappa }}_{\text{GLM}}\left(t\right)$ (solid red; see Eq 7), which takes into account the effect of the threshold-voltage coupling. **(E)-(F)** Same plots as in panel *D*, but for increased mean input *μ*_I_.

To answer this question, we analyzed the iGIF model response to three fluctuating currents with different offsets *μ*_I_ and a fixed standard deviation *σ*_I_ (Fig 7). In response to a weak input *μ*_I_ = 90 pA, the firing rate *f* ≈ 2 Hz was low and threshold movements induced by different action potentials did not accumulate significantly (Fig 7A, top). Given the modest contribution of spike-dependent threshold adaptation, spikes were initiated with relatively low firing thresholds and subthreshold membrane potential fluctuations were mostly confined to low voltages *V* < *V*_i_, where the coupling between firing threshold and subthreshold membrane potential is not active (Fig 7A, bottom). However, even at low firing rates, the threshold-voltage coupling was recruited during large positive fluctuations of the membrane potential (Fig 7A, top red). Increasing the input strength to *μ*_I_ = 230 pA resulted in an output firing rate of *f* ≈ 10 Hz (Fig 7B, top). In this regime, spikes were initiated at larger thresholds, subthreshold membrane fluctuations occurred at voltages *V* ≈ *V*_i_ (Fig 7B, bottom) and the nonlinear coupling between firing threshold and membrane potential was constantly active. Further increasing the mean input to *μ*_I_ = 450 pA made the iGIF model fire at *f* ≈ 18 Hz (Fig 7C). In this regime, threshold movements triggered by different spikes accumulated significantly making it possible for the subthreshold membrane potential to reach more depolarized voltages *V* > *V*_i_, where the threshold coupling reaches its maximal strength (Fig 7C, bottom).

Overall, the results reported in Fig 7 provide evidence for the existence of a non-trivial interplay between spike-dependent and voltage-dependent threshold movements. In particular we found that, at large firing rates, the increased contribution of spike-dependent adaptation allowed the subthreshold membrane potential to attain voltages at which the strength of the nonlinear threshold coupling is maximal (compare voltage distributions in Fig 7A–7C, bottom). Thus, increasing *μ*_I_ progressively strengthened the threshold sensitivity to subthreshold voltage fluctuations (compare red traces in Fig 7A–7C, top). As a result of the threshold dynamics, the membrane potential distribution always peaked below the average firing threshold (Fig 7A–7C, bottom), a characteristic signature of the subthreshold regime in which neurons are sensitive to rapid input fluctuations [54].

### The firing threshold dynamics adaptively control the effective timescale of somatic integration

To study the functional implications of the progressive activation of the coupling between firing threshold and membrane potential, we systematically reduced the iGIF model to a GLM (see Fig 2A and Materials and Methods). More precisely, we analytically computed the GLM filters ${\hat{\kappa }}_{\text{GLM}}\left(t\right)$ and ${\hat{h}}_{\text{GLM}}\left(t\right)$ that best approximate the iGIF model response to a set of stationary currents with different average intensities *μ*_I_ (see Materials and Methods). First, because of the spike-triggered conductance, the effective membrane filter $\kappa_{\text{m}}^{\text{eff}}\left(t\right)$ (cf, Eqs 28 and 29) becomes shorter with increasing *μ*_I_ (Fig 7D–7F black; see Materials and Methods). Second, because of the threshold-voltage coupling, the shape of the effective integration filter ${\hat{\kappa }}_{\text{GLM}}\left(t\right)$ is affected by the threshold dynamics according to the following equation:

$${\hat{\kappa }}_{\text{GLM}}\left(t\right)=\kappa_{\text{m}}^{\text{eff}}\left(t\right)-{\bar{G}}_{\theta }\cdot \int_{0}^{\infty }\frac{1}{\tau_{\theta }}\exp\left(-\frac{s}{\tau_{\theta }}\right)\cdot \kappa_{\text{m}}^{\text{eff}}\left(t-s\right)ds,$$ (7)

where ${\bar{G}}_{\theta }$ is the average activation level of the threshold-voltage coupling *G*_*θ*_(*V*) computed with respect to the voltage distribution (see Fig 7A–7C, bottom and Materials and Methods).

In response to a low input *μ*_I_, the strength of the threshold coupling was weak on average and somatic integration was mostly controlled by the effective membrane filter (Fig 7D). Increasing *μ*_I_ progressively shifted the membrane potential distribution towards voltages where the threshold coupling becomes more and more important (see Fig 7B and 7C). Consequently, the effective integration filter ${\hat{\kappa }}_{\text{GLM}}\left(t\right)$ became shorter than $\kappa_{\text{m}}^{\text{eff}}\left(t\right)$ (Fig 7E and 7F red). These theoretical results, whose accuracy was confirmed by extracting the effective integration filter *κ*_GLM_(*t*) directly from the spiking activity generated by the iGIF model (Fig 7D–7F gray), indicate that both the spike-triggered conductance and the firing threshold dynamics actively control the timescale of somatic integration. Importantly, since only the first mechanism affects the membrane voltage, the impact of the threshold dynamics on somatic integration cannot be seen in the subthreshold dynamics of the membrane fluctuations.

**Fig 8:**
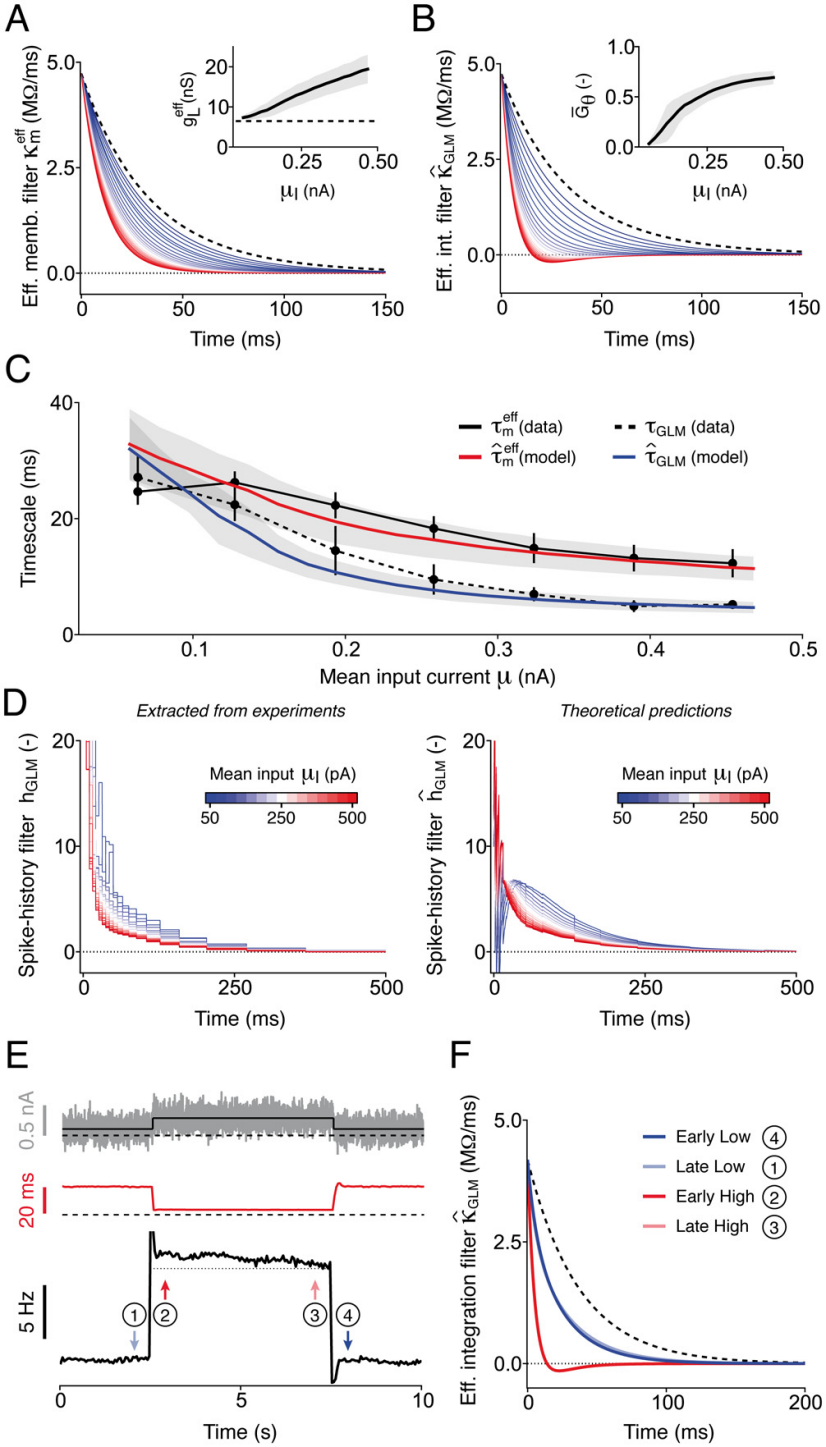
The iGIF model captures and explains complex forms of adaptation. **(A)** Average effective membrane filters $\kappa_{\text{m}}^{\text{eff}}\left(t\right)$ computed with iGIF model parameters extracted from 6 Pyr neurons by increasing *μ*_I_ from 0.05 nA (blue) to 0.5 nA (red, see colorbars in panel *D*). The passive membrane filter *κ*_m_(*t*) (dashed black) is shown for comparison. Inset: average effective conductance $g_{\text{L}}^{\text{eff}}$ as a function of *μ*_I_. The gray area indicates one standard deviation across neurons and the dashed black line indicates the passive leak conductance *g*_L_. **(B)** Same results as in panel *A*, but for the average integration filter ${\hat{\kappa }}_{\text{GLM}}\left(t\right)$. Inset: average coupling strength ${\bar{G}}_{\theta }$ as a function of the mean input *μ*_I_. Conventions are as in panel *A*. **(C)** The effective membrane timescale ${\hat{\tau }}_{\text{m}}^{\text{eff}}$ (red) and the effective timescale of integration ${\hat{\tau }}_{\text{GLM}}$ (blue) predicted by the iGIF model with parameters extracted from six neurons match the experimental data (black; copied from Fig 2D). Colored lines and gray areas indicate the mean and one standard deviation across neurons. The effective timescales of integration ${\hat{\tau }}_{\text{GLM}}$ (red) predicted by the iGIF model were obtained by fitting a single-exponential function to ${\hat{\kappa }}_{\text{GLM}}\left(t\right)$. **(D)** The iGIF model explains the adaptive changes in the spike-history filter *h*_GLM_(*t*) (see Fig 2C). Left: average spike-history filter *h*_GLM_(*t*) obtained by fitting a GLM to artificial data generated by simulating the iGIF model response to fluctuating currents of increasing *μ*_I_ (see colorbar). Right: average theoretical filters ${\hat{h}}_{\text{GLM}}\left(t\right)$ computed using iGIF model parameters extracted from 6 Pyr neurons. Because of the approximations involved in the analytical derivation of ${\hat{h}}_{\text{GLM}}\left(t\right)$, the strength of the GLM spike-history filters are underestimated during the firsts $\tau_{\text{m}}^{\text{eff}}$ ms (see Materials and Methods). **(E)-(F)** Switching experiment performed in a iGIF model (with parameters extracted from a typical cell) to study the temporal evolution of single neuron adaptation induced by a sudden change in *μ*_I_. **(E)** Top: fluctuating current (gray) generated by periodically switching *μ*_I_ (dark gray) between 0.1 nA and 0.27 nA, with cycle period *T*_cycle_ = 10 s (only one cycle is shown). Middle: effective timescale of integration ${\hat{\tau }}_{\text{GLM}}$ as a function of time. Bottom: output firing rate. While spike-frequency adaptation occurs on both fast and slow timescales, changes in ${\hat{\tau }}_{\text{GLM}}$ triggered by a switch in *μ*_I_ are almost instantaneous. Horizontal black lines indicate (from top to bottom): 0 nA, 0 ms and 0 Hz. **(F)** Comparison between effective integration filters ${\hat{\kappa }}_{\text{GLM}}\left(t\right)$ estimated at different moments in time during the switching experiment (see arrows in panel *E*). The filters estimated at steady-state (late low, late high; defined as the last 150 ms before the stimulus switch) closely resemble the ones estimated right after the stimulus switch (early low, early high; first 150 ms after the stimulus switch), indicating that adaptive changes in ${\hat{\kappa }}_{\text{GLM}}\left(t\right)$ are almost instantaneous. The passive membrane filter *κ*_m_(*t*) (dashed black) is shown for comparison. In all panels, input currents were generated according to Eq 8 with *σ*_I_ = 100 pA and *τ*_I_ = 3 ms.

Using the iGIF model parameters extracted from experimental data, we applied our theoretical results and systematically computed the effective membrane filter $\kappa_{\text{m}}^{\text{eff}}\left(t\right)$ (Fig 8A) and the effective integration filter ${\hat{\kappa }}_{\text{GLM}}\left(t\right)$ (Fig 8B) for different input strengths *μ*_I_. Notably, the timescales of the theoretical filters $\kappa_{\text{m}}^{\text{eff}}\left(t\right)$ and ${\hat{\kappa }}_{\text{GLM}}\left(t\right)$ explained the experimental discrepancy between the effective membrane timescale $\tau_{\text{m}}^{\text{eff}}$ and the effective timescale of somatic integration *τ*_GLM_ (Fig 8C). Finally, an interaction between the threshold-coupling and the adaptation current *I*_A_ captured by the iGIF model explained why the GLM spike-history filter *h*_GLM_(*t*) was shortened at increasing input strengths *μ*_I_ (Fig 8D; see Materials and Methods).

Overall, these results indicate that the iGIF model can be interpreted as an enhanced GLM in which both the input filter *κ*_GLM_(*t*) and spike-history filter *h*_GLM_(*t*) adapt to the input statistics.

### L5 Pyr neurons feature two distinct forms of adaptation

In order to study the temporal dynamics of single neuron adaptation, we finally performed a switching experiment in which the iGIF model was stimulated with a fluctuating current, whose mean *μ*_I_ periodically switched between a low and a high value, with cycle period *T*_cycle_ = 10 s (Fig 8E). In response to a sudden increase in *μ*_I_, the output firing rate transiently increased and then decayed over multiple timescales, confirming that in the iGIF model the combined action of the spike-triggered conductance and the spike-triggered movement of the firing threshold mediates spike-frequency adaptation. Similarly, in response to a sudden decrease of *μ*_I_, the output firing rate initially dropped and then partially recovered.

By computing ${\hat{\kappa }}_{\text{GLM}}\left(t\right)$ at different moments in time relative to the cycle, we found that, in contrast to spike-frequency adaptation, adaptive changes in the effective timescale of somatic integration ${\hat{\tau }}_{\text{GLM}}$ were almost instantaneous (Fig 8E, red and 8F). The reason for this is that the effects induced by the threshold coupling depends on the momentary voltage, rather than on the mean firing rate. The results presented in Fig 8E and 8F are reminiscent of the adaptive behavior previously observed in retinal ganglion cells [55] and in motion sensitive neurons [56] responding *in vivo* to external stimuli with varying statistics and show that an intrinsic nonlinearity, such as the one resulting from fast Na^+^-channel inactivation, can mediate a rapid and seemingly complex form of adaptation [16, 57].

Overall, our results indicate that L5 Pyr neurons respond to a sudden change in the input statistics by adapting both their output firing rate and the temporal window over which the input current is effectively integrated. The high speed at which the timescale of somatic integration adapts indicates that, regardless of the input statistics, L5 Pyr neurons respond preferentially to rapid input fluctuations resulting, e.g., from coincident spike arrival.

## Discussion

To study single-neuron computation over a broad range of input statistics, we stimulated L5 Pyr neurons with a set of *in vivo*-like fluctuating currents generated by independently varying the magnitude of its mean *μ*_I_ and standard deviation *σ*_I_. Confirming previous results from different cortical areas and species, we found that L5 Pyr neurons of the mouse SSC featured sensitivity to rapid input fluctuations independently of *μ*_I_ (Fig 1). To investigate the computational principles underlying enhanced sensitivity to rapid input fluctuations, we fitted several GLMs and found that the effective timescale of somatic integration was not constant, but shortened with increasing *μ*_I_ (Fig 2). Since this timescale reduction was not entirely explained by changes in the passive properties of the membrane (Fig 2D), we analyzed the voltages at which spikes originated and found that the spike threshold increased with *μ*_I_ and was reduced in the presence of input fluctuations (Fig 3).

To explain these experimental findings within a single mathematical framework and investigate the functional properties of spike-threshold adaptation, we introduced a new spiking model obtained by extending our previous GIF model [44, 45]. Fitting the iGIF model to data revealed that the firing threshold was nonlinearly coupled to the subthreshold membrane potential on a short timescale and was affected by previous spikes over multiple timescales (Fig 4). The iGIF model was able to capture the firing rate response of L5 Pyr neurons over broad range of input statistics and outperformed our previous GIF model [44, 45] in predicting the occurrence of individual spikes with millisecond precision (Figs 5 and 6). Analytically reducing the iGIF model to a GLM, finally showed that the nonlinear dynamics of the firing threshold adaptively shorten the effective timescale over which L5 Pyr neurons integrate their inputs, thus enhancing sensitivity to rapid input fluctuations over a broad range of input statistics (Figs 7 and 8).

### Spike-threshold variability

The membrane potential at which spikes are initiated is highly variable both *in vitro* and *in vivo* [24, 58, 59]. Many studies have demonstrated that the voltage threshold for spike initiation correlates not only with the average value of the membrane potential [43, 59], but also with the duration of previous interspike intervals [29, 41, 42, 44, 60] and with the speed at which the firing threshold is approached [42, 58, 59, 61]. When single neurons are stimulated with current ramps of different slopes, rapid rates of depolarization are often associated with lower thresholds [48, 62]. While in rat pyramidal neurons this phenomenon has been linked to the activation of Kv1 channels [48], those are apparently not expressed in L5 Pyr neurons of the mouse SSC [63, 64]. As indicated by theoretical studies based on the standard Hodgkin-Huxley model [20, 21] and by *in vivo* recordings from barn owl auditory neurons [24], spike-threshold sensitivity to the membrane depolarization rate can alternatively be mediated by a nonlinear coupling between firing threshold and membrane potential due to fast Na^+^-channel inactivation. Providing indirect evidence for this hypothesis, we found that the somatic voltage at which action potentials originated was highly variable, depended nonlinearly on the average membrane potential, was positively correlated with the DC component of the input current *μ*_I_ and decreased with increasing input fluctuations *σ*_I_ (Fig 3). Dual patch-clamp recordings have shown that the membrane potential recorded at the soma does not necessarily reflect the membrane potential at the axon initial segments (AIS), where spikes are initiated [65, 66]. These results questioned whether somatic threshold variability, and more generally the somatic spike shape, reflects real integrative properties of the neuron or are just an epiphenomenon of spike back-propagation from the AIS [66, 67], but see [21, 68–70].

To investigate the origin of spike-threshold variability, we fitted our intracellular recordings with a new spiking neuron model, in which the firing threshold is dynamically coupled to the membrane potential via a state variable *θ* and depends linearly on previous spikes (see Eqs 4 and 5). A spike-triggered movement of the firing threshold can in principle be implemented by incrementing the value of *θ* after the emission of each action potential [25]. However, the timescale over which spike-triggered effects occur can in principle differ from the timescale *τ*_*θ*_ of the threshold-voltage coupling. For this reason, our iGIF model accounts for spike-dependent threshold adaptation with an independent process *γ*(*t*). Importantly, the functional shape of *γ*(*t*) and that of the steady-state function *θ*_∞_(*V*) of the threshold-voltage coupling were not assumed *a priori*, but were extracted from intracellular recordings using a nonparametric maximum-likelihood procedure.

By fitting the iGIF model to data we found that spike initiation is characterized by several timescales. First, the firing threshold is nonlinearly coupled to the subthreshold voltage on a rapid timescale *τ*_*θ*_ ∈ [5, 15] ms. Second, spike emission leads to a quasi-instantaneous increase of the firing threshold. Third, the threshold increase triggered by a spike decays over multiple timescales. In agreement with the hypothesis that the coupling between firing threshold and membrane potential results from fast Na^+^-channel inactivation [21] and confirming the results from a previous study in which a similar model was shown to account for spike-threshold variability *in vivo* [24], we found that *θ*_∞_(*V*) was correctly described by a smooth rectifier function (Fig 4E). Moreover, the coupling timescale *τ*_*θ*_, the half-inactivation voltage *V*_i_ and the asymptotic slope of *θ*_∞_(*V*) were consistent with the biophysical features of Na^+^-channels expressed in central neurons [21]. Na^+^-channel inactivation also occurs on slow timescales [71–75]. Slow Na^+^-channel inactivation has been previously linked to enhanced sensitivity to rapid input fluctuations [15, 17] and, in our iGIF model, is phenomenologically captured by the spike-triggered component of the firing threshold dynamics. Confirming our earlier results [45] and consistent with the fact that spike-frequency adaptation does not have a preferred timescale [49], we indeed found that threshold movements induced by previous spikes lasted for several seconds and were characterized by a power-law decay (see Fig 4D).

Overall, as discussed in ref. [21] and reviewed in the Materials and Methods, the firing threshold dynamics featured by the iGIF model can be interpreted as phenomenological description of Na^+^-channels in which inactivation is independently controlled by one fast and several slow gating variables [15, 71]. While being inferred by maximizing spike-timing prediction (rather than by directly fitting the somatic voltage at spike onset), our model did not simply account for the spike-threshold dependence on input statistics (see Fig 5). Indeed, it also allowed us to: i) improve spike-timing prediction (Fig 6) and ii) explain why the effective timescale of somatic integration is not entirely controlled by the effective membrane timescale and adapts to the input statistics (see Fig 8). In line with the findings of Fontaine et al. [24], these results indicate that somatic spike-threshold variability is not a measurement artifact, but *a genuine feature of cortical action-potential generators* [68].

### Functional implications of spike-threshold adaptation

Comparing the GLM filters *κ*_GLM_(*t*) extracted by fitting the spiking responses to different current injections revealed that the effective timescale over which neurons integrate their inputs decreases at increasing input strengths *μ*_I_ (Fig 5D). Previous studies in which the response properties of spiking neurons have been analyzed using the Linear Nonlinear Poisson model [76] already suggested that the integration properties of single neurons—as measured by the spike-triggered average of the input [77]—depend on the input statistics (see, e.g., refs. [39, 78, 79]). However, since the spiking activity of single neurons responding *in vitro* to external currents is strongly non-Poissonian (see, e.g., ref. [39]), the STAs reported in the above-mentioned studies can not be directly interpreted as the temporal window over which the input current is somatically integrated. Most importantly, input-dependent changes in STA could merely reflect changes in spiking statistics, which are unrelated to changes in somatic integration [36–38]. In contrast to LNP models, GLMs account for non-Poissonian statistics by means of a specific spike-history filter *h*_GLM_(*t*). Consequently, the input filter *κ*_GLM_(*t*) can characterize changes in intrinsic neuronal properties which are separable from changes in spiking statistics.

To test whether the timescale reduction revealed by the GLM-based analysis was due to a conductance increase resulting, e.g., from the progressive recruitment of a subthreshold adaptation current [18, 19], we quantified the effect of *μ*_I_ on the effective membrane filter $\kappa_{\text{m}}^{\text{eff}}\left(t\right)$. While *κ*_GLM_(*t*), together with the spike-history filter *h*_GLM_(*t*), describes how the input current is transformed into a spiking probability, $\kappa_{\text{m}}^{\text{eff}}\left(t\right)$ transforms input currents into subthreshold membrane voltages. Although the membrane filters extracted from the data did shorten at increasing *μ*_I_, the membrane timescale reduction was not sufficient to explain the timescale reduction revealed by the GLM-based analysis (Fig 2D). This result demonstrates that *κ*_GLM_(*t*) does not simply reflect the subthreshold membrane response properties, but also accounts for additional mechanisms capable of regulating the effective timescale of integration without affecting the membrane voltage.

To elucidate this point and better understand the biophysical meaning of the GLM input filter *κ*_GLM_(*t*), we analytically reduced the iGIF model to a GLM [22, 23] and found that the effective timescale of somatic integration is controlled by: i) the ratio between the cell capacitance *C* and leak conductance *g*_L_, ii) the conductance-based spike-triggered adaptation *η*(*t*) and iii) the dynamic coupling *θ*(*t*) between firing threshold and membrane potential (see Eq 7). Importantly, while the first two neuronal features affect the subthreshold membrane potential and explain the observed membrane timescale reduction (see Fig 8A–8C), the threshold-voltage coupling only acts on the effective timescale of integration, thus explaining the experimental discrepancy between *τ*_GLM_ and $\tau_{\text{m}}^{\text{eff}}$ (Fig 8C).

The consequences of fast Na^+^-channel inactivation for threshold-voltage coupling and shortened effective timescale of somatic integration have been previously studied in refs. [21, 24]. Briefly, assuming that any postsynaptic spike has already been emitted and that the membrane potential is resting at *V*_0_, a presynaptic spike inducing a postsynaptic current *I*_EPSC_(*t*) = *ϵδ*(*t*) of weak amplitude *ϵ* will evoke an excitatory postsynaptic potential *δV*_EPSP_(*t*) = *ϵκ*_m_(*t*), where *κ*_m_(*t*) is the passive membrane filter. If the firing threshold is coupled to the membrane potential according to Eqs 4 and 5, this EPSP will in turn evoke a firing threshold increase $\delta V_{\text{T,EPSP}}\left(t\right)\approx G_{\theta }\left(V_{0}\right)\int_{0}^{t}\kappa_{\theta }\left(s\right)\delta V_{\text{EPSP}}\left(t-s\right)ds$, where $\kappa_{\theta }\left(t\right)=\frac{1}{\tau_{\theta }}\exp(-\frac{t}{\tau_{\theta }})$ is a low-pass filter with timescale *τ*_*θ*_ and the approximation comes from the fact that *θ*_∞_(*V*) has been linearized around *V*_0_ (see Eq 5). Since in our model the spike emission probability depends on the difference between membrane potential and firing threshold, increasing the firing threshold has the same effect as decreasing the membrane potential. Consequently, a model in which the firing threshold is coupled to the membrane potential can be seen as a model with constant firing threshold in which every EPSP is accompanied by an inhibitory postsynaptic potential *δV*_IPSP_(*t*) = *δV*_T, EPSP_(*t*) with characteristic rise time *τ*_*θ*_ [21]. In such a model, presynaptic spikes will thus be integrated via an effective postsynaptic potential *δV*_eff_(*t*) = *δV*_EPSP_(*t*)−*δV*_IPSP_(*t*) that decays on a shorter timescale compared to *δV*_EPSP_(*t*). Since the steady-state function *θ*_∞_(*V*) of the threshold-voltage coupling is well described by a smooth rectifier (see Fig 4E), the magnitude of *δV*_IPSP_(*t*) depends on the postsynaptic membrane potential *V*_0_ via a sigmoidal gain function $G_{\theta }\left(V\right)=\frac{d\theta_{\infty }}{dV}\left(V\right)$ (see Fig 7A–7C). When the postsynaptic potential *V*_0_ at spike arrival is smaller than the half-inactivation voltage *V*_i_, *G*_*θ*_(*V*) is small and the threshold increase (i.e., the inhibitory signal) becomes negligible. However, when *V*_0_ > *V*_i_, *G*_*θ*_(*V*) increases and the effective postsynaptic potential *δV*_eff_(*t*) shortens. The fact that the average subthreshold membrane potential increases with the input strength *μ*_I_ finally explains why strong inputs are effectively integrated on shorter timescales (Fig 7).

While confirming that fast Na^+^-channel inactivation enhances sensitivity to rapid inputs, our results indicate that slow Na^+^-channel inactivation acts as an homeostatic mechanisms by increasing the mean firing threshold in response to strong inputs. In agreement with the suggestion of Platkiewicz and Brette [21], we found that slow and fast Na^+^-channel inactivation interact in a nontrivial way. Indeed, by increasing the mean firing threshold, slow Na^+^-channel inactivation allows for subthreshold voltage fluctuations to occur at more depolarized voltages *V* ≫ *V*_i_, where *G*_*θ*_(*V*) is large (see Fig 4E).

Dynamic-clamp experiments have demonstrated that CA1 Pyr neurons operating in high-conductance state switch their behavior from integrators to differentiators [11, 78]. This complex form of adaptation has been qualitatively reproduced in a phase-plane model according to which a shunting-induced increase of the firing threshold allows for M-currents to activate at subthreshold voltages [78]. The interplay between shunting and subthreshold adaptation reported in ref. [78] shares some similarities with the nonlinear interaction we found between slow, spike-dependent and fast, voltage-dependent threshold adaptation. Two important differences are however to be noticed. First, the subthreshold adaptation process in ref. [78] results from the activation of an M current, which, in contrast to the threshold-voltage coupling featured by our iGIF model, modifies the spike response properties of the cell by altering the effective membrane filter $\kappa_{\text{m}}^{\text{eff}}\left(t\right)$. In agreement with our result that enhanced sensitivity to rapid input fluctuations is mediated by a dynamic coupling between firing threshold and membrane potential, direct measurements of the subthreshold membrane impedance of CA1 Pyr neurons [11] have shown that the differentiating behavior observed in high-conductance state is not mediated by subthreshold resonance [80]. Second, while in ref. [78] the threshold increase triggering the switch from temporal integration to differentiation is gated by the synaptic input (i.e., by the conductance increase resulting from synaptic bombardment), the recruitment of the threshold-voltage coupling in the iGIF model is gated by the postsynaptic firing rate via slow, spike-dependent threshold adaptation (see Fig 4E).

Overall, our results explain why L5 Pyr neurons maintain sensitivity to rapid input fluctuations regardless of their working regime, confirm theoretical predictions about the firing threshold dyanmics, demonstrate that the firing threshold plays a crucial role in determining the integration properties of single neurons, and shed new light on *in vivo* studies where sensitivity to rapid signals has been linked to the voltage threshold for spike initiation [58, 59, 61].

### Connection to sensory adaptation

The statistical properties of sensory stimuli (such as, e.g., visual inputs or whisker movements) are complex and vary over time. Given their limited dynamic ranges, sensory systems must thus constantly adapt their coding strategies in order to provide an efficient representation of the external world [81, 82]. Adaptation is a hallmark of virtually all sensory systems and occurs over multiple timescales. Many sensory systems adapt their input-output transformation not only to the mean, but also to the variance and even to higher-order moments of the input statistics [83].

Over the last decades, sensory adaptation has been repeatedly investigated using an experimental paradigm known as the *switching experiment* [83]. In a switching experiment, the spiking activity of a neuron is recorded *in vivo*, while the animal is presented with a controlled stimulus that rapidly fluctuates over time. To assess adaptation to local input statistics, the stimulus is generated according to a Gaussian process whose mean (or variance) is periodically switched between two values. In response to a sudden change in the variance (i.e., the contrast) of a visual input, both retinal ganglion cells and motion-sensitive neurons in the fly feature two forms of adaptation [55, 56]. Right after a change in input contrast, these neurons rapidly modify the shape of their receptive field, thereby adapting the stimulus feature to which they are preferentially responsive. While this mechanism is very fast, the same neurons also feature a slower form of adaptation that manifests itself in a decay of the output firing rate over multiple timescales. This second mechanism, known as spike-frequency adaptation, does not induce further changes in the receptive field, but simply reduces the overall excitability of the neuron. Since both the timescale and the net effect of these two adaptation processes are different, it has been hypothesized that changes in feature selectivity and output firing rate are controlled by two independent mechanisms [83]. Whether and how both forms of adaptation can be mediated by intrinsic neuronal properties remains unclear.

To investigate this issue, we performed a switching experiment *in silico* by presenting our iGIF model with an *in vivo*-like fluctuating current, whose mean *μ*_I_ periodically switched between two values (Fig 8E–F). Our results predict that, in response to a sudden change in the mean input, L5 Pyr neurons rapidly modify their *receptive field*, that is, the temporal window according to which the input current is somatically integrated. Moreover, the spiking response predicted by our iGIF model was characterized by spike-frequency adaptation that occurred on much slower timescales. Overall, our results suggest that the forms of sensory adaptation reported in refs. [55, 56] do not required network effects, but can in principle be implemented by two qualitatively distinct cellular mechanisms: a fast, nonlinear coupling between firing threshold and subthreshold voltage—possibly mediated by fast Na^+^-channel inactivation—and a slow, spike-triggered processes—possibly mediated by slow Na^+^-channel inactivation and by other ion-channels mediating afterhyperpolarization currents.

### Are simplified neuron models getting complicated?

Cortical neurons feature a strong nonlinear behavior, making single-neuron computation dependent on the input statistics [10–12, 39]. During the last decade, a number of simplified threshold models [22, 29, 41, 53, 84–86], including our previous GIF model [44, 45], have been shown to accurately predict the spiking response evoked *in vitro* by stationary (or quasi-stationary) currents [51, 87, 88]. Simplified threshold models are usually obtained by partially linearizing the dynamics of conductance-based biophysical models [21, 89, 90]. Thus, when assessed on a broad range of input statistics, their performance generally drops [14] (see also Fig 5). For the same reason, model parameters extracted from responses to different inputs generally differ [13] (see also Fig 2). These results reflect the inability of simplified threshold models of capturing the nonlinear dynamics underlying complex forms of single neuron adaptation. Overall, designing and fitting to data a simplified spiking model capable of predicting the electrical activity of cortical neurons operating in different regimes remains a big challenge [14]. Indeed, increasing the complexity of a spiking neuron model rapidly makes parameter estimation a difficult problem [91].

To solve this problem, we introduced the inactivating Generalized Integrate-and-Fire (iGIF) model which extends the standard Leaky Integrate-and-Fire model in three directions. First, noise is introduced by the escape-rate model for stochastic spike generation [22, 29, 46]. Second, a spike-triggered conductance *η*(*t*) [47] and a spike-triggered movement of the firing threshold *γ*(*t*) [85, 92, 93] are included for spike-frequency adaptation. Third, a nonlinear coupling *θ* between membrane potential and firing threshold [21, 25, 48] is added for enhanced sensitivity to input fluctuations [7].

The iGIF model can be related to a conductance-based model in which: i) the combined activity of voltage-activated and Ca^2+^-activated K^+^-channels [94–96] results in the spike-triggered conductance increase *η*(*t*) [47, 97], ii) Na^+^-channels are gated by a fast inactivation variable [98] implementing the nonlinear coupling *θ* between firing threshold and membrane potential [20, 21] and iii) Na^+^-channels are also gated by an additional set of slow inactivation variables [71, 99], whose combined activity results in a spike-triggered threshold movement *γ*(*t*) decaying over multiple timescales [20, 21]. While the biophysical interpretation of the spike-triggered conductance *η*(*t*) is well established (see, e.g., refs. [47, 97]), the link between the inactivation properties of Na^+^-channels and the firing threshold dynamics featured by the iGIF model is more involved and thus deferred to the Materials and Methods section. Would such a conductance-based model perform better than our iGIF model? Since fitting detailed biophysical models to electrophysiological data is cumbersome [51], especially in situations where the ion-channel dynamics are not known *a priori*, answering this question is not trivial.

In contrast to conductance-based models, the iGIF model accounts for the intricate dynamics of different ion-channels with simple phenomenological descriptions. Consequently, its parameters can be efficiently extracted from intracellular recordings with a new two-step procedure, which extends previous methods [22, 44, 45, 84]. In contrast to previous studies [27, 100], where optimal parameters of spiking neuron models have been inferred directly from the *f*-*μ*_I_ curves, our fitting procedure exploit the information contained in the subthreshold membrane potential fluctuations, thus allowing for the characterization of adaptation mechanisms resulting from the firing threshold dynamics [34]. Despite its relative simplicity, the iGIF model captures complex forms of single neuron adaptation, providing a good description of L5 Pyr neurons over an extended range of input statistics. Despite its relative complexity, the iGIF model can be analytically mapped to a GLM with input-dependent filters. We conclude that the iGIF model provides an accurate, yet intuitive description of single-neuron computation over a broad range of input statistics.

## Materials and Methods

### Electrophysiological recordings

All procedures in this study were conducted in conformity with the Swiss Welfare Act and the Swiss National Institutional Guidelines on Animal Experimentation for the ethical use of animals. The Swiss Cantonal Veterinary Office approved the project following an ethical review by the State Committee for Animal Experimentation.

Somatic whole-cell *in vitro* current clamp recordings were performed on 300 *μ*m thick parasagittal acute slices from the right hemispheres of male P13-P15 C57Bl/6J wild-type mice. Brains were quickly dissected and sliced (HR2 vibratome, Sigmann Elektronik, Germany) in ice-cold artificial cerebrospinal fluid (ACSF) (in mM: NaCl 124.0, KCl 2.50, MgCl_2_ 10.0, NaH_2_PO_4_ 1.25, CaCl_2_ 0.50, D-(+)-Glucose 25.00, NaHC0_3_ 25.00; pH 7.3, s.d. 0.1, aerated with 95% O_2_ / 5% CO_2_), followed by a 15 minute incubation at 34°C in standard ACSF (in mM: NaCl 124.0, KCl 2.50, MgCl_2_ 1.00, NaH_2_PO_4_ 1.25, CaCl_2_ 2.00, D-(+)-Glucose 25.00, NaHC0_3_ 25.00; pH 7.40, aerated with 95% O_2_ / 5% CO_2_). To ensure intact axonal and dendritic arborisation, electrophysiological recordings were conducted in slices cut parallel to the apical dendrites. Recordings in Layer 5 of the primary somatosensory cortex were performed at 32, s.d. 1°C in standard ACSF with an Axon Multiclamp 700B Amplifier (Molecular Devices, USA) using 5–7 M*Ω* borosilicate pipettes, containing (in mM): K^+^-gluconate 110.00, KCl 10.00, ATP-Mg^2+^ 4.00, Na_2_-phosphocreatine 10.00, GTP-Na^+^ 0.30, HEPES 10.00, biocytin 5.00 mg/ml; pH 7.30, 300 mOsm. Cells were visualized using infrared differential interference contrast video microscopy (VX55 camera, Till Photonics, Germany and BX51WI microscope, Olympus, Japan).

Data were acquired with sampling frequency Δ*T*^−1^ = 10 kHz using an ITC-18 digitizing board (InstruTECH, USA) controlled by a custom-written software module operating within IGOR Pro (Wavemetrics, USA). Voltage signals were low-pass filtered (Bessel, 10 kHz) and not corrected for the liquid junction potential of +14.5 mV estimated from Igor XOP Patcher’s Calculator (courtesy of Drs. F. Mendez and F. Würriehausen, MPI for Biophysical Chemistry, Göttingen, Germany). Consequently, the membrane potentials and the firing thresholds reported in this study are positively biased by 14.5 mV. Only cells with an access resistance ≤ 20 M*Ω* (17.7, s.d. 2.3 M*Ω*, n = 6) were retained for further analysis.

### Current injections

In all the experiments included in this study, neurons were stimulated with fluctuating currents *I*(*t*) generated according to an Ornstein-Uhlenbeck process:

$$\tau_{\text{I}}\dot{I}\left(t\right)=-I\left(t\right)+\mu_{\text{I}}+\sqrt{2\tau_{\text{I}}}\sigma_{\text{I}}\cdot \psi \left(t\right),$$ (8)

where *ψ*(*t*) is a Gaussian white-noise process with zero mean and unitary variance, *τ*_I_ is the correlation timescale, *μ*_I_ is the mean current and *σ*_I_ defines the magnitude of the fluctuations (that is, the standard deviation of the current). The temporal correlation of the input was fixed to *τ*_I_ = 3 ms and input currents *I*(*t*) were generated at a sampling rate Δ*T*^−1^ = 10 kHz.

To measure the impact of input fluctuations on the single-neuron input-output transfer function (i.e., the *f*-*μ*_I_ curve), we somatically injected a set of 5-second currents with different means *μ*_I_ and standard deviations *σ*_I_. To let the cell recover, injections were performed with interstimuli intervals of 25 seconds. Similar protocols have already been applied in previous studies [7, 8, 27]. Here, to broadly explore the parameter space (*μ*_I_, *σ*_I_) and to accurately estimate the experimental *f*-*μ*_I_ curves, we considered four different standard deviations *σ*_I_ ∈ {0, 50, 100, 150} pA and eight different means *μ*_I_ ∈ [0, *μ*_max_] nA, with *μ*_max_ begin cell-dependent. Each neuron was stimulated with 32 different inputs that were presented randomly. This protocol was repeated 3 times giving a total number of 96 current injections. When stimulated with strong inputs, pyramidal neurons undergo spike failure and can not sustain repetitive firing for long periods of time (see, e.g., ref. [71]). At the beginning of each experiment, the maximum current *μ*_max_ was defined in such a way as to reach saturation of the steady-state firing rate while preventing spike failures. For that, neurons were tested with 6-s-long noiseless currents (i.e., *σ*_I_ = 0) of increasing magnitude *μ*_I_. Cells that could not sustain continuous firing for a DC current *μ*_I_ < 0.4 nA were not further considered. The maximal mean input *μ*_max_ was comprised between 0.4 and 0.55 nA.

To evaluate model performance in predicting the occurrence of individual spikes, a different set of experiments was performed. Currents were generated according to Eq 8, but in this case, the stochastic process used to generate the input was made non-stationary by modulating the standard deviation *σ*_I_ with a sinusoidal function of time:

$$\sigma_{I}\left(t\right)=\sigma_{0}\left(1+\frac{1}{2}\cdot \sin\left(\frac{2\pi }{T}\cdot t\right)\right),$$ (9)

with *T* = 5 s being the modulation period. For each cell, input parameters were calibrated to obtain an average firing rate of 10 Hz oscillating between 7 and 13 Hz, approximatively. After calibration, input parameters were in the following ranges: *μ*_I_ ∈ [120, 190] pA, *σ*_0_ ∈ [120, 190] pA. Since the spiking responses of both neurons and GIF models are stochastic, spike-timing prediction was quantified on a *test set* obtained by 9 repetitive injections of the same (i.e. *frozen-noise*) 20-s current generated according to Eqs 8 and 9. For parameter extraction, a *training set* was used in which single neurons were stimulated with a single 120-s current having the same statistics as the *test set*, but in which a different realization of the white-noise process *ψ*(*t*) was used. All the injections were performed with inter-stimuli intervals of 25 seconds.

### Data preprocessing

When acquired with the same electrode used to inject the external input *I*(*t*), current-clamp recordings *V*_rec_(*t*) are biased versions of the membrane potential *V*_data_(*t*) [41]. This bias can in principle be removed using series resistance or bridge balance compensation. However, perfect calibration of these methods is technically difficult to achieve. Moreover, during long experiments, the electrode properties, and in particular the series resistance *R*_e_, are subject to change [45]. Quantitative comparison between membrane potentials evoked by input currents having different offsets *μ*_I_ requires accurate electrode compensation. Indeed, a non-neutralized series resistance ${\tilde{R}}_{\text{e}}$ would lead, on average, to a mean input-dependent bias $V_{\text{bias}}\left(\mu_{\text{I}}\right)={\tilde{R}}_{\text{e}}\mu_{\text{I}}$ (see, e.g., ref. [45]). To avoid this and others problems, for all the *in vitro* recordings included in this study, online series resistance compensation was complemented by Active Electrode Compensation (AEC) [41, 101]. For that, the same procedure applied as in ref. [45] was used. In case of long experiments, estimating the electrode properties at different moments in time can improve the quality of the data by removing drifts due to slow changes in the electrode properties [45]. For this reason, electrode filters used for AEC were extracted from 10-s subthreshold injections performed before the *training set*, before the *test set* and every sixteen injections in the protocol used to measure the *f*-*μ*_I_ curves. Subthreshold input currents were generated according to Eq 8 with *μ*_I_ = 0 nA, *σ*_I_ = 75 pA and *τ*_I_ = 3 ms.

### Extracting a voltage threshold for spike initiation from *in vitro* recordings

Following ref. [7], we estimated the voltage threshold for each spike in the dataset by measuring the membrane potential *V*_data_ at which the depolarization rate *dV*_data_/*dt* became larger than 10 mV/ms (see Fig 3E–3H). With this definition, threshold crossing always occurred less than 1 ms before the membrane potential reached 0 mV. Since at the soma spike initiation is very sharp (see ref. [69, 102] and Fig 3F and 3H) and since our analysis is only based on relative variations between the voltage threshold in different conditions, rather than on absolute values, the exact definition does not matter [20]. In Fig 3C and 3D, the average subthreshold membrane potential was computed by discarding all the data points $\left\{t|t\in \left[{\hat{t}}_{j}-2\;\text{ms},{\hat{t}}_{j}+10\;\text{ms}\right]\right\}$ that were too close to action potentials $\left\{{\hat{t}}_{j}\right\}$.

### Generalized Linear Model (GLM)

In the GLM [22, 23], spikes are generated stochastically with firing intensity λ_GLM_(*t*) defined as:

$$\lambda_{\text{GLM}}\left(t\right)=\lambda_{0}\cdot \exp\left(\int_{0}^{\infty }\kappa_{\text{GLM}}\left(s\right)I\left(t-s\right)ds+\sum_{{\hat{t}}_{j}<t}h_{\text{GLM}}\left(t-{\hat{t}}_{j}\right)\right),$$ (10)

where λ_0_ is a constant, *κ*_GLM_(*t*) is an arbitrarily-shaped filter through which the input current is effectively integrated and *h*_GLM_(*t*) accounts for all spike-triggered processes that make the single-neuron activity history-dependent [46]. GLM parameter extraction is performed using the standard maximum likelihood method described in refs. [22, 23]. For that, both *κ*_GLM_(*t*) and *h*_GLM_(*t*) were expanded in linear combinations of rectangular basis functions. In the main text, the GLM filters analytically derived from the iGIF model (see below) are denoted ${\hat{\kappa }}_{\text{GLM}}\left(t\right)$ and ${\hat{h}}_{\text{GLM}}\left(t\right)$.

### Inactivating Generalized Integrate-and-Fire model (iGIF)

In the iGIF model, spikes are produced stochastically according to the conditional firing intensity λ(*t*) defined by the exponential escape-rate model [29, 46]:

$$\lambda \left(t\right)=\lambda_{0}\exp\left(\frac{V\left(t\right)-V_{T}\left(t\right)}{\Delta V}\right),$$ (11)

where *V*(*t*) is the membrane potential, *V*_T_(*t*) is a dynamic threshold, Δ*V* defines the level of stochasticity and, without loss of flexibility, we fixed λ_0_ = Δ*T*^−1^ = 10 kHz. In the limit Δ*V* → 0, the iGIF model becomes deterministic and action potentials are fired reliably each time $\hat{t}$ the firing threshold is reached (i.e., when $V\left(\hat{t}\right)=V_{\text{T}}\left(\hat{t}\right)$). When Δ*V* > 0, the iGIF model is stochastic, and the probability of emitting an action potential at time $\hat{t}\in \left[t,t+dt\right]$ is given by [6]:

$$p\left(\hat{t}\in \left[t,t+dt\right]\right)=1-\exp\left(-\int_{t}^{t+dt}\lambda \left(s\right)ds\right),$$ (12)

meaning that, when *V* = *V*_T_, a spike is emitted during a time step of Δ*T* = 0.1 ms with probability *p* = 0.63. When Δ*V* > 0, spikes can be emitted even if the membrane potential is lower than the firing threshold. Similarly, the membrane potential can cross the firing threshold without evoking a spike. With increasing Δ*V*, spike emission becomes more stochastic and progressively loses sensitivity to *V*(*t*)−*V*_T_(*t*). The maximal level of stochasticity is reached when Δ*V* → ∞. In this limit, the iGIF model becomes formally equivalent to an homogeneous Poisson process with stochastic intensity λ_0_. In the iGIF model, probabilistic spike emission is the only source of stochasticity. Importantly, the level of stochasticity Δ*V* is not assumed *a priori*, but is extracted from experimental data along with all the other parameters (see below).

The dynamics of the subthreshold membrane potential are modeled as a leaky integrator augmented with a spike-triggered conductance *η*(*t*) that describes the time course of the conductance change after a spike. More precisely, the membrane potential evolves according to the following differential equation:

$$C\dot{V}=-g_{\text{L}}\left(V-E_{\text{L}}\right)+I-\sum_{{\hat{t}}_{j}<t}\eta \left(t-{\hat{t}}_{j}\right)\cdot \left(V-E_{\text{R}}\right),$$ (13)

where *C*, *g*_L_ and *E*_L_ describe the passive properties of the membrane, *τ*_m_ = *C*/*g*_L_ is the passive membrane timescale, $\left\{{\hat{t}}_{1},{\hat{t}}_{2},{\hat{t}}_{3},\ldots \right\}$ are the spike times, *E*_R_ is a reversal potential and *I* is the external input. Conductance changes triggered by different spikes accumulate and produce spike-frequency adaptation (or facilitation). The functional shape of *η*(*t*) is not assumed *a priori*, but is extracted from experimental data (see below). After each spike, the membrane potential is reset to *V*_reset_ and the numerical integration only restarts after an absolute refractory period *T*_ref_. The absolute refractory period was set to *T*_ref_ = 4 ms and the voltage reset was estimated by computing the average membrane potential after a spike (i.e. $V_{\text{reset}}={\langle V\left({\hat{t}}_{j}+T_{\text{ref}}\right)\rangle }_{j}$). Since a period of absolute refractoriness can also be implemented by setting the first milliseconds of the spike-triggered threshold movement to high values, the particular choice of *T*_ref_ is not crucial.

The dynamics of the firing threshold *V*_T_ are given by:

$$V_{\text{T}}\left(t\right)=\theta \left(t\right)+\sum_{{\hat{t}}_{j}<t}\gamma \left(t-{\hat{t}}_{j}\right),$$ (14)

where *γ*(*t*) describes the movement of the firing threshold after the emission of an action potential. Similar to *η*(*t*), the functional shape of *γ*(*t*) is not assumed *a priori*, but is extracted from the data. Since *γ*(*t*) can only account for spike-dependent effects, the model is augmented with an additional state variable *θ*(*t*), which couples the firing threshold to the subthreshold membrane potential. Based on theoretical results obtained by a systematic reduction of the Hodgkin-Huxley model (see next section), this coupling is expected to be nonlinear [20]. In the iGIF model, the dynamics of *θ*(*t*) are given by

$$\tau_{\theta }\dot{\theta }=-\theta +V_{\text{T}}^{*}+\theta_{\infty }\left(V\right),$$ (15)

where $V_{\text{T}}^{*}$ is a constant, *τ*_*θ*_ is the characteristic timescale on which the threshold reacts to changes in the membrane potential and *θ*_∞_(*V*) is the voltage-dependent steady-state towards which *θ* converges. To avoid *a priori* assumptions on the biophysical processes underlying the threshold-voltage coupling, *θ*_∞_(*V*) is defined in the iGIF-free model as an arbitrary function of the membrane potential and is extracted from experimental data using a novel non-parametric maximum likelihood approach (see below). Spike-dependent movements of the firing threshold are modeled by *γ*(*t*) and the state variable *θ* is reset to $V_{\text{T}}^{*}$ after each spike.

### iGIF-Na model

The iGIF-Na model is defined exactly as the iGIF-free model except for the fact that the dynamics of *θ*(*t*) are predefined based on the biophysics of Na^+^-channels [21] and is given by Eqs 5 and 6. The reasons for this choice are reviewed now.

#### Biophysical interpretation of the threshold-voltage coupling

In the standard Hodgkin-Huxlely (HH) model, the sodium current *I*_Na_ responsible for spike initiation is gated by two indepenent variables, *m* and *h*, that describe Na^+^-channel activation and inactivation, respectively [98]. Na^+^-channel activation occurs on very short timescales and can therefore be considered as instantaneous [20, 41, 89]. At spike onset, *I*_Na_ is thus well approximated by an exponential function of the membrane potential $I_{\text{Na}}\propto h\exp(\frac{V-V_{\text{T}}^{*}}{k_{\text{a}}})=\exp(\frac{V-(V_{\text{T}}^{*}-k_{\text{a}}\log h)}{k_{\text{a}}})$, where $V_{\text{T}}^{*}$ is a constant, *k*_a_ is a biophysical parameter describing the sharpness of Na^+^-channel activation and $\theta =V_{\text{T}}^{*}-k_{a}\log h$ defines a smooth threshold for spike initiation [20]. Since in the HH model Na^+^-channel inactivation follows a first-order kinetics $\tau_{\text{h}}\left(V\right)\dot{h}=-h+h_{\infty }\left(V\right)$, the firing threshold *θ* is coupled to the membrane potential according to the following differential equation [21]:

$$\tau_{\theta }\dot{\theta }=-\theta +V_{\text{T}}^{*}+\theta_{\infty }\left(V\right),$$ (16)

with *θ*_∞_(*V*) = −*k*_a_ log *h*_∞_(*V*) and *τ*_*θ*_ = 〈*τ*_h_(*V*)〉_*P*(*V*)_. In cases where the steady-state inactivation curve *h*_∞_(*V*) is correctly described by an inverse sigmoidal function $h_{\infty }\left(V\right)={\left(1+\exp(\frac{V-V_{\text{i}}}{k_{i}})\right)}^{-1}$, the effective coupling between firing threshold and membrane potential is characterized by the smooth rectifier function $\theta_{\infty }^{\text{Na}}\left(V\right)$ featured by the iGIF-Na model and defined in Eq 6.

The dynamics described by Eq 16 are reminiscent of that of the adaptation variable *ω* in the Adaptive Exponential Integrate-and-Fire model (ADEX) [103]. However, while *ω* describes a subthreshold current and depends linearly on *V*, *θ* describes the firing threshold dynamics and depends nonlinearly on *V*.

#### Biophysical interpretation of spike-triggered threshold movements

As shown in refs. [20, 21], Eq 16 provides a good approximation of the firing threshold dynamics whenever |*θ*(*t*)−*θ*_∞_(*V*(*t*))|≪*k*_a_. While this condition is generally satisfied, during the emission of an action potential the membrane potential rises almost instantaneously to a large voltage *V*_AP_, where *h*_∞_(*V*_AP_)≈0. Consequently, *θ*_∞_ becomes much larger than *θ*, making Eq 16 inadequate to describe spike-dependent threshold movements resulting from Na^+^-channel inactivation. In simplified neuron models that do not describe the voltage dynamics during action potentials, spike-triggered effects can however be accounted for by resetting the state variable *θ* → *θ*+Δ*θ* after the emission of each spike [20, 21]. This update rule is mathematically equivalent to an exponential spike-triggered threshold movement *γ*_fast_(*t*) = Δ*θ* exp(−*t*/*τ*_*θ*_), where the magnitude of the threshold jump Δ*θ* = *k*_a_ Δ*T*_AP_/*τ*_h_(*V*_AP_) is obtained by considering that during the duration Δ*t*_AP_ of an action potential, the inactivation variable *h* evolves approximately according to $\tau_{\text{h}}\left(V_{\text{AP}}\right)\dot{h}=-h+h_{\infty }\left(V_{\text{AP}}\right)$ and thus decreases by a fixed amount $\Delta h=h\left(\hat{t}\right)\left(1-\exp\left(-\Delta t_{\text{AP}}/\tau_{\text{h}}\left(V_{\text{AP}}\right)\right)\right)$, with $h(\hat{t})$ being the inactivation level at spike onset [20, 21].

Previous experimental studies have shown that Na^+^-channels are also characterized by slow inactivation [71–75]. In conductance-based models, slow Na^+^-channel inactivation is generally described by an additional set of slow gating variables *s*_*i*_ that independently control the sodium conductance [71]. In such a model, the sodium current at spike onset is well approximated by $I_{\text{Na}}\propto \prod_{i=1}^{n}s_{i}\cdot h\exp(\frac{V-V_{\text{T}}^{*}}{k_{\text{a}}})=\exp(\frac{V-(\theta +\sum_{i=1}^{n}\phi_{\text{i}})}{k_{\text{a}}})$, where *φ*_i_ = −*k*_a_ log *s*_i_, the dynamics of *θ* are as in Eq 16 and the smooth threshold for spike initiation is given by [21]:

$$V_{\text{T}}=\theta +\sum_{i=1}^{n}\varphi_{\text{i}}.$$ (17)

Following the same reasoning as for fast Na^+^-channel inactivation, one can show that if i) the dynamics of each slow inactivation variable *s*_i_ are given by $\tau_{s_{i}}{\dot{s}}_{\text{i}}=-s_{i}+s_{\text{i},\infty }\left(V\right)$ and ii) slow inactivation is only recruited during action potentials (i.e., the steady-state functions *s*_i,∞_(*V*) are such that *s*_i,∞_(*V*)<1 only for very depolarized potentials), then each gating variable *s*_i_ mediates a spike-dependent threshold modulation $\phi_{\text{i}}\left(t\right)=\sum_{\hat{t}}\gamma_{i}\left(t-\hat{t}\right)$, with *γ*_i_(*t*) ∝ exp(−*t*/*τ*_*s*_*i*__). Plugging this result into Eq 17 leads to the threshold dynamics featured by the iGIF-Na model:

$$V_{\text{T}}\left(t\right)=\theta \left(t\right)+\sum_{\hat{t}}\gamma \left(t-\hat{t}\right),$$ (18)

where the spike-triggered threshold movement $\gamma \left(t\right)=\gamma_{\text{fast}}\left(t\right)+\sum_{\text{i = 1}}^{n}\gamma_{i}\left(t\right)$ combines the spike-triggered effects mediated by the inactivation variables {*h*, *s*_1_,…, *s*_n_} and thus decays over multiple timescales {*τ*_*θ*_, *τ*_*s*_1__,…, *τ*_*s*_*n*__}. More details of these derivations can be found in refs. [20, 21].

### iGIF model parameter extraction

#### Fitting procedure for the iGIF-free model

Given the input current *I*(*t*), the intracellular membrane potential *V*_data_(*t*), its first-order derivative ${\dot{V}}_{\text{data}}\left(t\right)=\left[V_{\text{data}}\left(t+\Delta T\right)-V_{\text{data}}\left(t\right)\right]/\Delta T$ and the experimental spike train $\left\{{\hat{t}}_{j}\right\}$, iGIF model parameters are obtained with a new two-step procedure developed by extending the methods introduced in refs. [44, 45]. A Python implementation of the fitting procedure is freely available at https://github.com/pozzorin/GIFFittingToolbox.

In the first step, all the parameters describing the subthreshold dynamics are extracted by minimizing the sum of squared errors between the voltage derivative observed in the experiment and the one predicted by the model (see Eq 13). To allow for convex optimization and avoid *a priori* assumptions on the timescales of adaptation, the spike-triggered conductance was expanded in a linear combination of basis functions $\eta \left(t\right)=\sum_{i=1}^{K}\eta_{i}b_{i}^{\left(\eta \right)}\left(t\right)$, where $\left\{b_{i}^{\left(\eta \right)}\left(t\right)\right\}$ is a set of *K* = 40 log-spaced non-overlapping rectangular functions and the parameters {*η*_*i*_} define the shape of *η*(*t*). The least-square estimate of the subthreshold parameters $\beta_{\text{sub}}^{T}\left(E_{\text{R}}\right)=C^{-1}\cdot \left[g_{\text{L}},E_{\text{L}}g_{\text{L}},\eta_{1},...,\eta_{K},1\right]$ is obtained by solving a multilinear regression problem [84]:

$${\hat{\beta }}_{\text{sub}}\left(E_{\text{R}}\right)={\left(X^{T}X\right)}^{-1}X^{T}{\dot{V}}_{\text{data}},$$ (19)

where *X* is a matrix made of vectors $X_{t}^{T}\left(E_{\text{R}}\right)$, which depends on *E*_R_ and are defined as:

$$X_{t}^{T}\left(E_{\text{R}}\right)= [-V_{\text{data}}\left(t\right),1,\sum_{j}b_{1}^{\left(\eta \right)}\left(t-{\hat{t}}_{j}\right)\left(V_{\text{data}}-E_{\text{R}}\right),...,\sum_{j}b_{\text{K}}^{\left(\eta \right)}\left(t-{\hat{t}}_{j}\right)\left(V_{\text{data}}-E_{\text{R}}\right),I\left(t\right)],$$ (20)

and ${\dot{V}}_{\text{data}}$ is a vector containing the membrane potential first-order derivative. Since the model does not capture the voltage trajectory during a spike, all the data points close to action potentials $\left\{t|t\in [{\hat{t}}_{j}\;-\;4\text{ ms};{\hat{t}}_{j}+T_{\text{ref}}]\right\}$ were excluded from the fit. As in ref. [44], the optimal reversal potential ${\hat{E}}_{\text{R}}$ (defined as the *E*_R_ minimizing the residuals of the regression in Eq 19) is extracted by an exhaustive search on the interval [-100, -40] mV.

In the second step, an estimate of the subthreshold membrane potential $\hat{V}\left(t\right)$ is obtained by numerically solving Eq 13. The threshold parameters are extracted by extending our previous maximum-likelihood approach [44, 45]. Again, to avoid *a priori* assumptions on the timescales of spike-dependent adaptation and on the shape of the coupling between firing threshold and subthreshold membrane potential, the two functions *γ*(*t*) and *θ*_∞_(*V*) were expanded in linear combinations of non-overlapping rectangular basis functions $\gamma \left(t\right)=\sum_{i=1}^{K}\gamma_{i}b_{i}^{\left(\gamma \right)}\left(t\right)$ and $\theta_{\infty }\left(V\right)=\sum_{i=1}^{M}\theta_{i}b_{i}^{\left(\theta \right)}\left(V\right)$. For the spike-triggered movement of the firing threshold *γ*(*t*), the same log-spaced rectangular functions already used for *η*(*t*) were chosen. For *θ*_∞_(*V*), *M* = 11 regularly spaced rectangular functions $\left\{b_{i}^{\left(\theta \right)}\left(V\right)\right\}$ were chosen that covered the interval of voltages $\left[\min_{j}\left\{\hat{V}\left({\hat{t}}_{j}\right)\right\},\max_{j}\left\{\hat{V}\left({\hat{t}}_{j}\right)\right\}\right]$ at which action potentials were initiated. Consequently, after integration of Eq 15, the time-dependent voltage threshold is given by

$$V_{\text{T}}\left(t\right)=V_{\text{T}}^{*}+\sum_{i=1}^{K}\gamma_{i}\cdot \sum_{{\hat{t}}_{j}<t}b_{i}^{\left(\gamma \right)}\left(t-{\hat{t}}_{j}\right)+\sum_{i=1}^{M}\theta_{i}\cdot f_{i}\left(t;\tau_{\theta }\right),$$ (21)

with $f_{i}\left(t;\tau_{\theta }\right)=\int_{0}^{t-{\hat{t}}_{\text{last}}}\tau_{\theta }^{-1}e^{-\frac{s}{\tau_{\theta }}}\cdot b_{i}^{\left(\theta \right)}\left(V\left(t-s\right)\right)ds$ and ${\hat{t}}_{\text{last}}$ denoting the time of the last spike before time *t*. With the exponential function in Eq 11, and assuming that the timescale *τ*_*θ*_ is known, the model log-likelihood is a convex function of the threshold parameters $\beta_{\text{th}}^{\text{T}}=\Delta V^{-1}\cdot \left[1,V_{\text{T}}^{*},\gamma_{1},\ldots ,\gamma_{K},\theta_{1},\ldots ,\theta_{M}\right]$ and can be written as follows [104]:

$$L\left(\beta_{\text{th}};\tau_{\theta }\right)=\log p\left(\left\{{\hat{t}}_{j}\right\}|\hat{V}\left(t\right);\beta_{\text{th}},\tau_{\theta }\right)=\sum_{t\in \{{\hat{t}}_{j}\}}Y_{\text{t}}\left(\tau_{\theta }\right)\cdot \beta_{\text{th}}-\sum_{t\in \Omega }\exp\left(Y_{\text{t}}\left(\tau_{\theta }\right)\cdot \beta_{\text{th}}\right),$$ (22)

with $\Omega =\{t|t\notin \left[{\hat{t}}_{j},{\hat{t}}_{j}+T_{\text{ref}}\right]\}$ being a set that excludes all the points falling in the period of absolute refractoriness and *Y*_t_(*τ*_*θ*_) being a vector of observables that implicitly depends on the parameter *τ*_*θ*_:

$$Y_{\text{t}}\left(\tau_{\theta }\right)= [\hat{V}\left(t\right),-1,-\sum_{j}b_{1}^{\left(\gamma \right)}\left(t-{\hat{t}}_{j}\right),\cdots ,-\sum_{j}b_{K}^{\left(\gamma \right)}\left(t-{\hat{t}}_{j}\right),-f_{1}\left(t;\tau_{\theta }\right),\cdots ,-f_{\text{M}}\left(t;\tau_{\theta }\right)].$$ (23)

Given *τ*_*θ*_, the maximum likelihood estimate of the other threshold parameters ${\hat{\beta }}_{\text{th}}\left(\tau_{\theta }\right)$ is obtained as in refs. [44, 45] by maximizing Eq 22 with standard gradient-ascent methods

$${\hat{\beta }}_{\text{th}}\left(\tau_{\theta }\right)=\underset{\beta_{\text{th}}}{argmax}\{L\left(\beta_{\text{th}};\tau_{\theta }\right)\}.$$ (24)

The optimal timescale of the coupling between threshold and membrane voltage ${\hat{\tau }}_{\theta }=\underset{\tau_{\theta }}{\mathrm{argmax}}\left\{L\left({\hat{\beta }}_{\text{th}}\left(\tau_{\theta }\right);\tau_{\theta }\right)\right\}$ is in turn obtained by systematically searching in the range *τ*_*θ*_ ∈ [0.5, 15] ms the value for which the log-likelihood is maximized. Despite the absence of a proof of joint convexity, the landscape of the log-likelihood function $L({\hat{\beta }}_{\text{th}}\left(\tau_{\theta }\right);\tau_{\theta })$ was smooth in *τ*_*θ*_ and always featured a unique maximum in the explored range (see Fig 4F).

#### Fitting procedure for the iGIF-Na model

The iGIF-Na model parameters were extracted from experimental data using a maximum likelihood approach closely resembling the nonparametric method described in the previous section. Briefly, the log-likelihood $L(\beta_{\text{th}}^{\text{Na}};\tau_{\theta },k_{\text{i}},V_{\text{i}})$ of the iGIF-Na model is convex in $\beta_{\text{th}}^{\text{Na}}=\Delta V^{-1}\cdot \left[1,V_{\text{T}}^{*},\gamma_{1},\ldots ,\gamma_{K},k_{\text{a}}\right]$. Consequently, given the nonlinear parameters *k*_i_, *V*_i_ and *τ_θ_*, all the other threshold parameters can be easily extracted by solving a convex optimization problem:

$${\hat{\beta }}_{\text{th}}^{\text{Na}}\left(\tau_{\theta },k_{\text{i}},V_{\text{i}}\right)=\underset{\beta_{\text{th}}^{\text{Na}}}{argmax}\{L\left(\beta_{\text{th}}^{\text{Na}};\tau_{\theta },k_{\text{i}},V_{\text{i}}\right)\}.$$ (25)

On the other hand, extracting the optimal parameters ${\hat{k}}_{\text{i}}$, ${\hat{V}}_{\text{i}}$ and ${\hat{\tau }}_{\theta }$ requires the solution of the following nonlinear optimization problem:

$$\left({\hat{k}}_{\text{i}},{\hat{V}}_{\text{i}},{\hat{\tau }}_{\theta }\right)=\underset{\left(\tau_{\theta },k_{\text{i}},V_{\text{i}}\right)}{argmax}\{L\left({\hat{\beta }}_{\text{th}}^{\text{Na}}\left(\tau_{\theta },k_{\text{i}},V_{\text{i}}\right);\tau_{\theta },k_{\text{i}},V_{\text{i}}\right)\}.$$ (26)

Performing an exhaustive search on a three-dimensional space is possible. Model parameters were however extracted by first fixing the coupling timescale *τ*_*θ*_ to the optimal value previously obtained by fitting the iGIF-free model and then performing an exhaustive search for *k*_i_ and *V*_i_ (see Fig 4G).

### Extracting the effective membrane timescale from subthreshold membrane potential

In order to extract from experimental data the effective membrane timescale $\tau_{\text{m}}^{\text{eff}}$ (see Fig 2D), intracellular recordings were split in different datasets according to *μ*_I_ and independently fitted with a Leaky Integrate-and-Fire model equipped with a spike-triggered current $\eta_{\text{C}}\left(t-{\hat{t}}_{\text{j}}\right)$, as opposed to a spike-triggered conductance $\eta (t-{\hat{t}}_{\text{j}})$. In other words, we used a model obtained by dropping the term (*V*−*E*_R_) from Eq 13 and replacing *g*_L_ by an effective conductance ${\tilde{g}}_{\text{L}}^{\text{eff}}$ and replacing *η*(*t*) by *η*_C_(*t*). By fitting this model to data using a linear regression similar to Eq 19, the average conductance increase mediated by spike-dependent processes is included in the effective leak conductance ${\tilde{g}}_{\text{L}}^{\text{eff}}$. The effective conductance-induced membrane timescale is thus given by $\tau_{\text{m}}^{\text{eff}}=C/{\tilde{g}}_{\text{L}}^{\text{eff}}$. In the main text, the effective membrane timescale analytically derived from the iGIF model (see below) is denoted ${\hat{\tau }}_{\text{m}}^{\text{eff}}$.

### iGIF model reduction

In order to understand how different adaptation processes captured by the iGIF model affect single-neuron computation, we systematically reduced the iGIF model to a GLM. The reduction is computed in three steps.

#### Step 1: Integral equation for the subthreshold membrane potential dynamics

The subthreshold dynamics of the iGIF model feature a spike-triggered conductance. Following ref. [28], Eq 13 can, however, be approximated by a leaky integrator equipped with a spike-triggered current *η*_C_(*t*):

$$C\dot{V}\left(t\right)=-g_{\text{L}}^{\text{eff}}\left(V\left(t\right)-\bar{V}\right)+\left(I\left(t\right)-\mu_{\text{I}}\right)-\left(\sum_{{\hat{t}}_{j}}\eta_{\text{C}}\left(t-{\hat{t}}_{\text{j}}\right)-{\bar{I}}_{\text{A}}\right),$$ (27)

where the effective conductance $g_{\text{L}}^{\text{eff}}=g_{\text{L}}+{\bar{g}}_{\eta }$ accounts for both the passive leak *g*_L_ and the average contribution of the spike-triggered conductance ${\bar{g}}_{\eta }=T^{-1}\cdot \int_{0}^{T}\sum_{{\hat{t}}_{\text{j}}}\eta \left(t-{\hat{t}}_{\text{j}}\right)dt$ during a long observation time *T*, *μ*_I_ is the mean input current, $\bar{V}=(g_{\text{L}}E_{\text{L}}+{\bar{g}}_{\eta }E_{\text{R}}+\mu_{\text{I}})/g_{\text{L}}^{\text{eff}}$ is the average membrane potential, ${\bar{I}}_{\text{A}}={\bar{g}}_{\eta }\left(\bar{V}-E_{\text{R}}\right)$ is the average current mediated by the spike-triggered conductance, and the spike-triggered current is given by $\eta_{\text{C}}(t)=\eta (t)\cdot (\bar{V}\;-\;E_{\text{R}})$.

The subthreshold dynamics of the membrane potential defined in Eq 27 can be rewritten in its integral form as [6]:

$$V\left(t\right)=\bar{V}+\frac{{\bar{I}}_{\text{A}}-\mu_{\text{I}}}{g_{\text{L}}}+\int_{0}^{\infty }\kappa_{\text{m}}^{\text{eff}}\left(s\right)I\left(t-s\right)ds-\sum_{{\hat{t}}_{j}<t}\eta_{\text{V}}\left(t-{\hat{t}}_{j}\right),$$ (28)

where $\kappa_{\text{m}}^{\text{eff}}\left(t\right)$ is the effective membrane filter defined as an exponential function

$$\kappa_{\text{m}}^{\text{eff}}\left(t\right)=\frac{1}{C}\exp\left(-\frac{t}{\tau_{\text{m}}^{\text{eff}}}\right)$$ (29)

with effective conductance-reduced membrane timescale $\tau_{\text{m}}^{\text{eff}}=C/g_{\text{L}}^{\text{eff}}$ and

$$\eta_{\text{V}}\left(t\right)=\int_{0}^{\infty }\kappa_{\text{m}}^{\text{eff}}\left(s\right) [\eta_{\text{C}}\left(t-s\right)+C\left({\bar{V}}_{\text{spikes}}-V_{\text{reset}}\right)\delta \left(t-s\right)]ds$$ (30)

describes the influence of both the spike-triggered current *η*_C_(*t*) and the spike-triggered reset upon the membrane potential. Here, ${\bar{V}}_{\text{spikes}}={\langle V\left({\hat{t}}_{\text{j}}\right)\rangle }_{\text{j}}$ is the average value of *V*(*t*) at spike times $\left\{{\hat{t}}_{\text{j}}\right\}$.

Since in this derivation the correlations between membrane potential fluctuations and conductance fluctuations have been neglected, *η*_V_(*t*) underestimates the real voltage change induced by the spike-triggered conductance during the first $\tau_{\text{m}}^{\text{eff}}$ milliseconds after a spike (see Fig 8C). The reason of this inaccuracy is that, right after the emission of an action potential, the total conductance of the iGIF model is, on average, larger than the effective conductance $g_{\text{L}}^{\text{eff}}$ used in Eq 29. Consequently, in the iGIF model, the early part of the adaptation current mediated by the spike-triggered conductance *η*(*t*) is transformed into a stereotypical voltage drop (i.e., an early AHP) by an integration filter which is on average faster than $\tau_{\text{m}}^{\text{eff}}$.

#### Step 2: Integral equation for the linearized firing threshold dynamics

We simplified the iGIF model threshold dynamics (i.e., Eq 5) by taking the first-order approximation $\theta_{\infty }^{\text{Na}}\left(V\right)\approx {\bar{C}}_{\theta }+{\bar{G}}_{\theta }\cdot V$, with ${\bar{C}}_{\theta }=\int_{-\infty }^{\infty }\theta_{\infty }^{\text{Na}}\left(V\right)P\left(V\right)dV-{\bar{G}}_{\theta }\bar{V}$ being a constant and ${\bar{G}}_{\theta }$ being the average of threshold-coupling gain $G_{\theta }\left(V\right)=\frac{d}{dV}\theta_{\infty }^{\text{Na}}\left(V\right)$ computed with respect to the membrane potential distribution *P*(*V*) (see Fig 7A–7C):

$${\bar{G}}_{\theta }=\int_{-\infty }^{\infty }G_{\theta }\left(V\right)P\left(V\right)dV.$$ (31)

After this first-order approximation, integrating Eq 15 over time results in:

$$V_{T}\left(t\right)=V_{T}^{*}+{\bar{C}}_{\theta }+{\bar{G}}_{\theta }\cdot \overset{\infty }{\underset{0}{\int }}\kappa_{\theta }\left(s\right)V\left(t-s\right)ds+\underset{{\hat{t}}_{j}<t}{\sum }\gamma_{\text{tot}}\left(t-{\hat{t}}_{j}\right),$$ (32)

where $\kappa_{\theta }\left(t\right)=\frac{1}{\tau_{\theta }}\exp(-\frac{t}{\tau_{\theta }})$ is the threshold-coupling filter. The spike-triggered filter $\gamma_{\text{tot}}\left(t\right)=\gamma \left(t\right)-\left({\bar{\theta }}_{\text{spike}}-V_{\text{T}}^{*}\right)\tau_{\theta }\kappa_{\theta }\left(t\right)$ combines the effects of both the spike-triggered threshold *γ*(*t*) and the spike-triggered reset of the threshold $\theta \to V_{\text{T}}^{*}$, with ${\bar{\theta }}_{\text{spike}}={\langle \theta ({\hat{t}}_{\text{j}})\rangle }_{\text{j}}$ being the average value of *θ*(*t*) at spike times $\left\{{\hat{t}}_{\text{j}}\right\}$.

#### Step 3: Mapping the iGIF model to the GLM

In the iGIF model, the spiking probability depends on the difference between membrane potential and firing threshold (see Eq 11). Thus, the different terms appearing in the integral Eqs 28 and 32 can be combined to obtain a GLM describing the firing intensity λ_lin_(*t*) of the linearized iGIF model:

$$\lambda_{\text{lin}}\left(t\right)=\lambda_{0}\cdot \exp\left(\frac{\int_{-\infty }^{\infty }{\hat{\kappa }}_{\text{GLM}}\left(s\right)I\left(t-s\right)ds+\sum_{{\hat{t}}_{j}<t}{\hat{h}}_{\text{GLM}}\left(t-{\hat{t}}_{j}\right)}{\Delta V}\right),$$ (33)

where all the constants have been absorbed in λ_0_, ${\hat{\kappa }}_{\text{GLM}}\left(t\right)$ is the effective integration filter defined as [21]:

$${\hat{\kappa }}_{\text{GLM}}\left(t\right)=\int_{-\infty }^{\infty } [\delta \left(s\right)-{\bar{G}}_{\theta }\kappa_{\theta }\left(s\right)]\kappa_{\text{m}}^{\text{eff}}\left(t-s\right)ds,$$ (34)

and ${\hat{h}}_{\text{GLM}}\left(t\right)$ is an effective spike-history filter that phenomenologically accounts for all the spike-triggered mechanisms in the iGIF model:

$${\hat{h}}_{\text{GLM}}\left(t\right)=\gamma_{\text{tot}}\left(t\right)+\int_{-\infty }^{\infty } [\delta \left(s\right)-{\bar{G}}_{\theta }\kappa_{\theta }\left(s\right)]\eta_{\text{V}}\left(t-s\right)ds.$$ (35)

In the absence of a coupling between firing threshold and membrane potential (i.e., when ${\bar{G}}_{\theta }=0$), somatic integration is entirely controlled by the effective membrane filter $\kappa_{\text{m}}^{\text{eff}}\left(t\right)$ (see Eq 34). In the presence of a threshold-voltage coupling (i.e., when ${\bar{G}}_{\theta }>0$), a running average is subtracted (see Eq 34), and the temporal window over which single neurons effectively integrate their inputs is shortened.

The effect of the firing threshold dynamics on somatic integration depends on the membrane potential distribution via the average coupling strength ${\bar{G}}_{\theta }$ (see Eqs 31 and 34). Since ${\bar{G}}_{\theta }$ increases with *μ*_I_ (see Fig 8B), augmenting the DC component of the input current reduces the effective timescale of somatic integration. Similarly, Eq 35 explains why increasing *μ*_I_ results in a shrinkage of the spike-history filter *h*_GLM_(*t*) (see Figs 2C and 8C).

### Performance evaluation

To avoid problems related to *overfitting* and to allow for a comparison between models that differ in the total number of parameters, the performance reported in this study were, unless specified otherwise, evaluated on separate data sets that were not used for parameter extraction. A quantitative measure of the quality of both the GIF and the iGIF model is provided by the log-likelihood [23]:

$$LL_{\text{model}}=\sum_{t\in \{\hat{t}\}}\log\lambda_{\text{model}}\left(t\right)-\int_{0}^{T}\lambda_{\text{model}}\left(t\right)dt$$ (36)

where λ_model_(*t*) is the conditional firing intensity of the model after parameter optimization, $\left\{\hat{t}\right\}$ is the experimental spike train and *T* is the total duration of the experiment on which the model performance were evaluated. All of the log-likelihoods reported in this study were normalized with respect to a homogenous Poisson process with constant intensity defined by the experimental firing rate $\bar{r}=N_{\text{spikes}}/T$, as well as with respect to the total number of spikes *N*_spikes_ [23]:

$$LL=\frac{1}{\log\left(2\right)\cdot N_{\text{spikes}}}\left(LL_{\text{model}}-N_{\text{spikes}}\left(\log\bar{r}-1\right)\right),$$ (37)

such that units are in bit per spike.

Spike-timing prediction was quantified using the spike-train similarity measure $M_{\text{d}}^{*}$ [105]. As in our previous studies [44, 45], $M_{\text{d}}^{*}$ was computed using the Kistler coincidence window with a temporal granularity of Δ = ±4 ms.
